# Ni/Pd-catalyzed Suzuki–Miyaura cross-coupling of alcohols and aldehydes and C–N cross-coupling of nitro and amines *via* domino redox reactions: base-free, hydride acceptor-free†

**DOI:** 10.1039/d0ra08344e

**Published:** 2020-12-10

**Authors:** Milad Kazemnejadi, Rebin Omer Ahmed, Boshra Mahmoudi

**Affiliations:** Department of Chemistry, College of Science, Shiraz University Shiraz 7194684795 Iran miladkazemnejad@yahoo.com; Anwar Shekha Medical City Sulaymaniyah Zip code 46024 Iraq; Research Center, Sulaimani Polytechnic University Sulaimani Iraq

## Abstract

Domino oxidation-Suzuki–Miyaura cross-coupling of benzyl alcohols with phenylboronic acid and domino reduction-C–N cross-coupling of the nitro compounds with aryl halides were carried out using a strong Ni/Pd bimetallic redox catalyst. The catalyst bearing a copolymer with two Ni/Pd coordinated metals in porphyrin (derived from demetalated chlorophyll b) and salen-type ligands, and pyridine moiety as a base functionality all immobilized on magnetite NPs was synthesised and characterized. The domino oxidation cross-coupling reaction was accomplished under molecular O_2_ in the absence of any hydride acceptor or/and base. Also, the domino reduction C–N cross-coupling reaction was performed in the presence of NaBH_4_ without the need for any base and co-reductant. This multifunctional catalyst gave moderate to good yields for both coupling reactions with high chemoselectivity. A wide investigation was conducted to determine its mechanism and chemoselectivity.

## Abstract 2

A new Ni/Pd bimetallic multifunctional catalytic system has been developed for the domino Suzuki–Miyaura cross-coupling of benzyl alcohols with phenyl boronic acid and domino reduction C–N cross-coupling of the nitro compounds with aryl halides.

## Graphical abstract

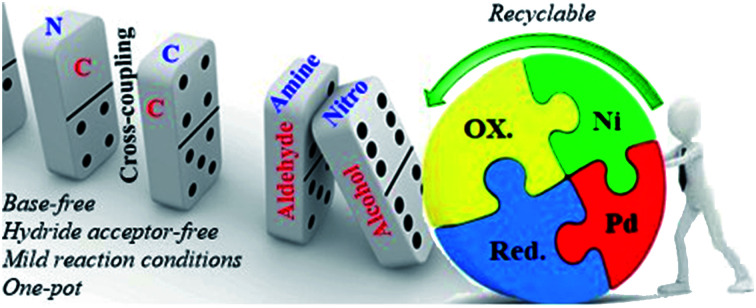

## Introduction

Cascade or domino reactions are one of the most applicable organic reactions, wherein consecutive C–C bond formation occurs in one step to prepare a complex molecule, and thus multiple chemical transformations are catalyzed by a single catalyst. Thus, they save energy, eliminate the troublesome work-up, reduce the generation of waste, increase synthetic efficiency, and are environmentally friendly and atom economical in most cases.^1^ These systems have prominent application in total synthesis, especially for the preparation of complex molecular structures and chiral cyclic derivatives from simple and readily available starting materials.^2^ For example, very recently, Chen *et al.* developed the domino 10-step total synthesis of FR252921 (complex macrocyclic immunosuppressants), and based on this domino synthesis, 14 biologically active compounds were synthesized.^3^ This is a good example of the preparation of very complex multi-step compounds that are very difficult or impossible to prepare through step-by-step and conventional methods.

The construction of eight-membered cyclic diaryl sulfides *via* the domino reaction of arynes with thioaurone analogues,^4^ toluene oxidation–Knoevenagel-condensation domino reaction,^5^ Cu-catalyzed aryl-I bond thiolation for the switchable synthesis of 2,3-dihydrobenzothiazinones and benzoisothiazolones,^6^ Cu-catalyzed one-pot synthesis of C-4 sulfonated isoquinolin-1(2*H*)-ones,^7^ and Co-catalyzed diastereoselective difluoroalkylation/Giese addition domino reactions^8^ are some of the recently reported applications of the domino approach in organic synthesis. Previously, Chandra *et al.* reviewed the application of domino reactions in catalytic C–C bond formation.^1^

An intelligent strategy for the synthesis of multistep compounds is the use of multifunctional catalysts. Climent *et al.* showed that the one-pot domino reactions catalyzed by multifunctional catalysts provide higher selectivity by adjusting the relative rates of the various successive steps in some cases.^9^

**Scheme 1:**
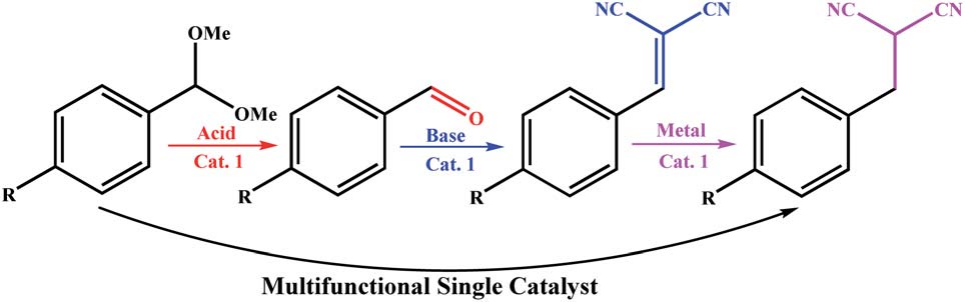
Development of a trifunctional catalytic system for an auto-tandem reaction by Ke *et al.*^10^

Different catalytic sites can be combined into a single catalyst for a specific purpose, and like a machine, perform a multi-step synthesis in one step. One of the best examples of these systems was reported by Ke *et al.* recently,^10^ where a multistep auto-tandem reaction was performed by an integrated-trifunctional single catalyst with acid, base and anchored Pd sites (Scheme 1). As shown in Scheme 1, the synthesis required three different types of catalysts with different nature in the stepwise traditional system; however they performed the synthesis using a multifunctional single catalyst.

In the last decade, multifunctional catalytic systems have been widely developed in organic synthesis^11,12^ as follows: (1) Pd-DTP@ZIF-8 in one-pot synthesis of 3-phenyl propyl benzoate,^13^ (2) HRh(CO)(PPh_3_)_3_ impregnated on Mg_1−*x*_Al_*x*_(OH_2_)^*x*+^(CO_3_^2−^)_*x*/*n*_·*m*H_2_O for the one-pot multistep synthesis of C_8_ aldehydes and alcohol from propene,^14^ (3) Pd-supported alkaline earth oxides and mixed oxides for the production of a series of fine chemicals involving one-pot multi-step reactions,^9^ (4) Au/Hap for the direct tandem synthesis of imines and oximes,^15^ (5) Fe_3_O_4_@MS–NH_2_@Pd for the direct synthesis of α-alkylated nitriles through facile one-pot multistep domino reaction sequences,^16^ and (6) Cs–Pr–Me–Cu(ii)–Fe_3_O_4_ for the cascade oxidation of benzyl alcohols/Knoevenagel condensation.^17^ The development of these catalytic systems creates milder conditions, reduces the synthesis steps in multi-step syntheses, synergistic effects in reactions, removes some additives, uses less catalyst, *etc.* Recently, bimetallic catalytic systems with different functions have attracted much attention. Nasseri *et al.* reported a Co–Cu bimetallic nanocatalyst with a synergistic and bifunctional performance for base-free Suzuki, Sonogashira, and C–N cross-coupling reactions.^11^

C–C and C–N cross-coupling reactions are of great importance due to their application in the construction of applicable biologically active molecules, functional materials, and pharmaceuticals.^18,19^ Thus, due to their inevitable application in the synthesis of organic and pharmaceutical molecules, the construction of these bonds is very important.^11,19^

Conventional C–C cross-coupling reactions such as Suzuki, Heck, and Sonogashira couplings utilize aryl halides as the electrophile, which in most cases is limited to aryl iodide and aryl bromide, and undesirable efficiency is obtained for aryl chlorides. In addition, these compounds are expensive, and their variety is limited due to their low availability. Therefore, using more affordable, inexpensive, and highly diversified alternatives to aryl halides as electrophiles will be beneficial to develop these reactions.

**Scheme 2:**
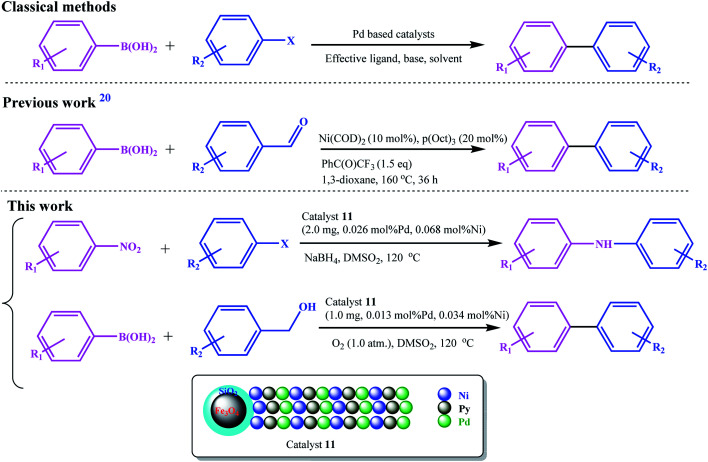
Comparison between the present work with the classical and previously (decarbonylative arylation reaction) reported protocol for C–C cross-coupling reactions.

Recently, Guo *et al.* developed an Ni-catalyzed decarbonylative arylation reaction of aldehydes with boronic esters (Scheme 2).^20^ The use of the inexpensive, more accessible, and high diversity aldehydes than aryl halides was the prominent advantage of their work. Accordingly, the development of these unconventional coupling electrophiles can be a revolution in the synthesis of medicinal and biological compounds and the study and discovery of new compounds.

We went a step further, using nitro and alcohol raw materials to develop this by designing a redox multifunctional bimetallic catalytic system. In this work, for the first time, the domino coupling of benzyl alcohols (or aldehydes) was performed by developing a three-functional catalyst including Pd/Ni centers and Py moieties (as a base functionality), with redox activity. Due to the different nature of oxidation and reduction reactions with coupling reactions, the most challenging part of this work was the design of the catalyst. Using two different ligand systems, Pd and Ni were coordinated to the ligands in two different steps. The base character was also given to the catalyst by grafting vinyl pyridine to the copolymer chain, and all these functionalities were combined on a single catalyst. The catalyst was designed in such a way that all reactions were performed using a very small amount of catalyst in the absence of any additives and base.

The presence of oxidation and reduction reagents did not disrupt the domino one-pot coupling reactions. In addition, due to the nature of the catalyst and the presence of Ni centers, the domino reaction of reduction-coupling of nitro compounds with aryl halides was also performed using this catalytic system in the presence of NaBH_4_.

## Results and discussion

**Scheme 3:**
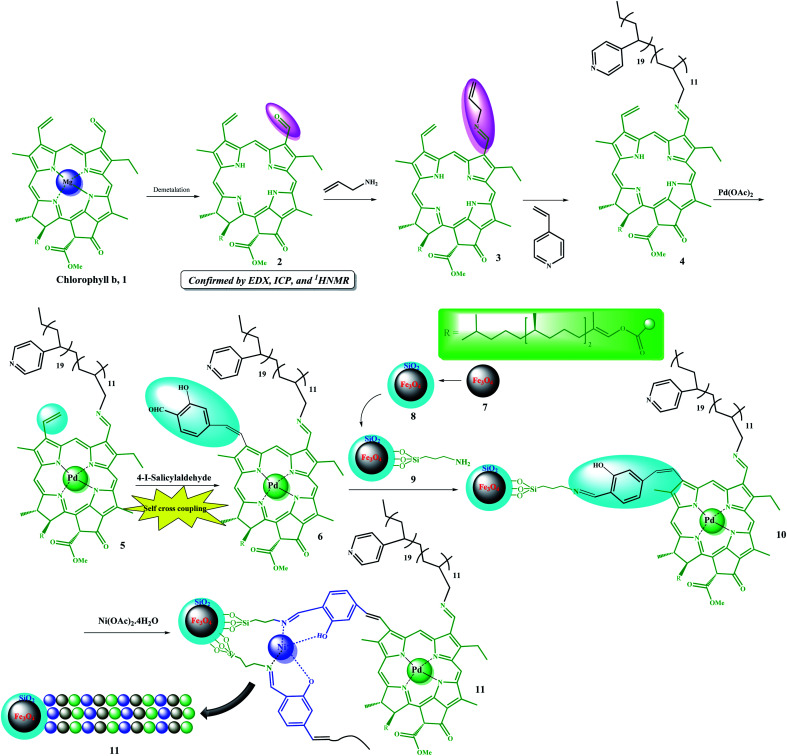
Step-by-step schematic showing the preparation of the Fe_3_O_4_@SiO_2_/(Py)-*copolymer*-(chlorophyll b) Ni/Pd (11) catalyst.

The multifunctional catalyst was prepared in several steps. As shown in Scheme 3, after extraction of chlorophyll b, it was demetalated by HCl (ESI, Fig. S1–S5†) and then allylated by allylamine for the subsequent copolymerization reaction, which was performed in the presence of 4-vinyl pyridine. Initially, Pd metal was coordinated to porphyrin groups in the chlorophyll. Due to the presence of a vinyl group and coordinated Pd in the structure of 5, it was auto-catalytically coupled to 4-iodo-salicylaldehyde by autocatalytic coupling reaction in the absence of any base. The resulting copolymer was then immobilized on Fe_3_O_4_@SiO_2_–NH_2_ magnetic nanoparticles by forming an iminium bond. The resulting catalyst possessed a salen-like ligand position, which could be used to coordinate another metal, and thus, catalyst 11 was produced by coordinating Ni ions to these groups. Catalyst 11 was well characterized by some analyses (ESI, Fig. S6–S9 and Table S1†). The average molecular weight of the copolymer was studied by GPC analysis.^6,21^ According to the GPC results, the average molecular weight of compound 4 was found to be 12 580.

Table 1 shows the results of the EDX elemental analysis and GPC for all the prepared compounds.

**Table 1:** Elemental and GPC analyses of compounds 1–11

Compound	EDX analysis (wt%)								AM_w_^a^
	C	O	N	Mg	Fe	Si	Pd	Ni	
1	78.60	10.76	6.66	3.98	—	—	—	—	—
2	80.75	11.74	7.51	—	—	—	—	—	884
3	81.39	9.35	9.26	—	—	—	—	—	924
4	81.09	8.80	10.11	—	—	—	—	—	12 580
5	79.88	7.64	9.08	—	—	—	3.40	—	—
6	80.00	7.88	9.08	—	—	—	3.04	—	—
10	26.54	31.12	6.22	—	22.84	11.55	1.73	—	—
11	26.11	30.53	6.08	—	22.55	11.30	1.41	2.02	—
12	81.45	9.43	9.12	—	—	—	—	—	11 440
13	79.87	8.91	8.06	—	—	—	3.16	—	—

a Average molecular weight based on GPC analysis.

**Scheme 4:**
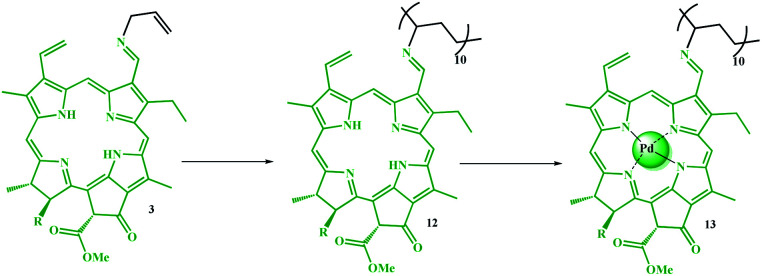
Preparation of polyvinyl chlorophyll-Pd complex (13).

The degree of polymerization (DP) of pyridine in copolymer 4 was determined by titration with acetic acid. This observed acidic property is related to the Py groups plus the pyrrole groups in the porphyrin ring. Therefore, to relate the degree of polymerization to the acidic nature of the Py groups, a homologue of 12 (Scheme 4) was prepared using the same procedure and almost the same degree of polymerization, and an acid test was performed on it. Thus, the results obtained from the acidic nature of compound 4 were subtracted from compound 12. It should be noted that this is only an approximation of the degree of polymerization for the catalyst since some of the acid may be used to hydrolyze the ester groups in the chlorophyll groups of 11 or 12. According to the titration results, the degree of polymerization for the pyridine in 4 is 19.

Thus, by correlating the GPC and acid-titration test results (as well as the EDX results), it can be concluded that the average degree of polymerization for chlorophyll in the copolymer loaded on the catalyst is equal to 11 (12 580 − 19(105) = 10 048/907 = 11). On the other hand, the acid titration test was reasonable proof for the successful grafting of Py monomer to the copolymer chain by the radical polymerization.

ICP analyses of catalyst 11 shows that 1.46 wt% Pd and 2.08 wt% Ni were present in its framework, completely in agreement with the EDX analysis.

### Optimization of reaction parameters

To perform coupling reactions through nitro and alcohol substrates, first the reaction parameters were studied to the achieve optimal conditions. The reaction parameters were studied for two reactions: (1) the oxidation-coupling reaction of benzyl alcohol with phenylboronic acid and (2) reduction-coupling reaction of nitrobenzene with iodobenzene, as model reactions. The results are summarized in Table 2, and entry 11 shows the optimal conditions for both reactions.

**Table 2:** Optimization of the catalytic oxidation of benzyl alcohol and reduction of nitrobenzene using 10 and 11, respectively

Entry	Solvent	C–C Suzuki coupling^a^			C–N coupling^b^			
		*T* (°C)	Cat. 11 (mg)	Yield (%)	*T* (°C)	NaBH_4_ (mmol)	Cat. 11 (mg)	Yield (%)
1	EtOH	Ref.	1.0	55	Ref.	2.0	2.0	50
2	MeOH	Ref.	1.0	60	Ref.	2.0	2.0	50
3	THF	Ref.	1.0	N.R.	Ref.	2.0	2.0	N.R.
4	CH_3_CN	Ref.	1.0	55	Ref.	2.0	2.0	40
5	DMF	Ref.	1.0	80	Ref.	2.0	2.0	88
6	Glycerol	120	1.0	84	160	2.0	2.0	85
7	Dioxane	Ref.	1.0	30	Ref.	2.0	2.0	45
8	Toluene	Ref.	1.0	55	Ref.	2.0	2.0	60
9	H_2_O	Ref.	1.0	45	Ref.	2.0	2.0	30
10	DMSO	Ref.	1.0	80	Ref.	2.0	2.0	90
**11**	**DMSO**_**2**_	**120**	**1.0**	**84**	**120** c	**2.0**	**2.0**	**90** d
12	DMSO_2_	80	1.0	44	80	2.0	2.0	60
13	DMSO_2_	160	1.0	85	160	2.0	2.0	90
14	DMSO_2_	R.T.	1.0	N.R.	Ref.	1.0	2.0	33
15	DMSO_2_	Ref.	0.001	50	Ref.	1.5	2.0	55
16	DMSO_2_	Ref.	0.05	68	Ref.	2.0	0.5	65
17	DMSO_2_	Ref.	1.5	80	Ref.	2.0	1.0	80
18	DMSO_2_	Ref.	2.0	84	Ref.	2.0	2.5	90
19^e^	DMSO_2_	120	1.0	30	120	0	2.0	N.R.

a Reaction conditions: benzyl alcohol (1.0 mmol), catalyst 11, temperature, solvent (2.0 mL, DMSO_2_: 3.36 g), O_2_ balloon (∼1.0 atm). For a better comparison, a constant time of 33 h was reported for all entries.

b Reaction conditions: nitrobenzene (1.0 mmol), NaBH_4_ (2.0 mmol), catalyst 11, solvent (2.0 mL, DMSO_2_: 3.36 g). For the better comparison, a constant time of 4.2 h was reported for all entries.

c No improvement was observed until 40 h.

d No improvement was observed until 8 h.

e The reaction was performed under air conditions.

The best solvent for both reactions was dimethyl sulfone (DMSO_2_), which at 120 °C produced 84% and 90% efficiencies for the domino Suzuki and C–N coupling reactions, respectively (Table 2, entry 11). DMSO_2_ has previously been identified as a green solvent in coupling reactions.^21^ Toluene, glycerol and DMSO also provided satisfactory efficiencies for both reactions, but due to environmental considerations and availability, DMSO_2_ was used as the optimal solvent for the coupling reactions. The optimum catalyst loading for the domino Suzuki C–C and C–N coupling reactions were 1.0 and 2.0 mg, respectively. Larger amounts of catalyst had no effect on the efficiency of the reactions, and at lower values, the efficiency decreased linearly (Table 2, entries 15–18).

The oxidation-coupling reaction was performed in the presence of molecular oxygen. The reaction under an air atmosphere produced only 30% efficiency for 33 h, indicating the importance of molecular O_2_ in the reaction. In addition, the catalyst showed excellent activity and selectivity for the oxidation of 1°-type alcohols to aldehydes in the presence of molecular oxygen, and thus can also be used as an effective catalyst for the selective oxidation of alcohol (ESI, Table S2†).

A reduction-coupling reaction was performed in the presence of 2 mmol NaBH_4_ (optimal value) (Table 2, entry 11). The reaction did not progress in the absence of NaBH_4_ (Table 2, entry 19), which shows that: (1) the coupling reaction occurs only through the amine precursor and (2) the reduction reaction is well performed by the catalyst in the presence of 2 mmol NaBH_4_.

The catalytic activity of 11 was evaluated for domino-Suzuki and C–N coupling reactions. The results of the domino Suzuki coupling reactions using alcohol or aldehyde in the presence of phenylboronic acid catalyzed by 11 are summarized in Table 3. The prominent advantages of the catalyst are the *in situ* oxidation of alcohol to aldehyde and its coupling with the phenyl ring.

**Table 3:** Ni/Pd-catalyzed C–C cross-coupling reaction from phenylboronic acid and aldehydes^a^

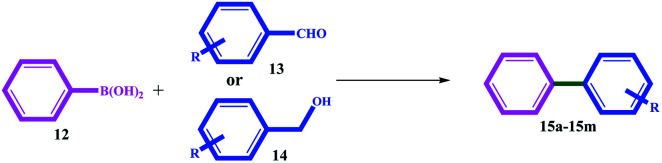						
Entry	R (alcohol or aldehyde)	Product	Time (h)		Yield^b^ (%)	
			From alcohol^a^^,^^c^	From aldehyde	From alcohol	From aldehyde
1	H	15a	33	30	84	84
2	4-MeO	15b	36	34	77	79
3	4-Me	15c	36	34	73	75
4	2-Me	15d	32	30	75	75
5	4-CN	15e	28	28	66	60
6	4-NO_2_	15f	30	30	74	77
7	1-Naphthyl	15g	40	36	70	74
8	4-Cl	15h	35	33	25^d^	35^e^
9	2-MeO	15i	36	35	70	70
10	4-NO_2_, 2-Me	15j	40	36	78	75
11	Nicotine	15k	28	24	80	82
12	Picoline	15l	28	26	82	80
13	2-Furfuryl	15m	40	35	65	80

a Reaction conditions: aldehyde or benzyl alcohol (1.0 mmol), phenylboronic acid (1.0 mmol), catalyst 11 (1.0 mg, 0.013 mol% Pd, 0.034 mol% Ni), DMSO_2_ (3.36 g, 35.7 mmol), and 120 °C.

b Isolated yield.

c O_2_ balloon (∼1.0 atm).

d 45% coupling product was takes place through Cl (major product).

e 33% coupling product was takes place through Cl (major product).

All the alcohol and aldehyde substrates were successfully coupled to the phenyl ring. Similar efficiencies were obtained for both the alcohol and aldehyde substrates, but longer times (between 2 and 5 h) were necessary for alcohols, which can be attributed to the time required for alcohol oxidation to aldehyde.

In general, aryl halides with electron withdrawing groups produced better efficiency (in terms of time and efficiency) than aryl halides with electron donor groups (for example, compare 19b with 19e, 19j with 19m, and 19s with 19u). On the other hand, electron donor substituents on the amines increased the coupling reaction efficiency. For example, 19f with similar hydroxyl substitution produced better efficiency than 19b, also 19c with 19g, and 19e with 19h. The leaving group also had an undeniable role in the productivity of the coupling reactions, and thus that the order of I > Br > Cl activity was quite evident for all the substrates (Table 4).

**Table 4:** Ni/Pd-catalyzed C–N cross-coupling reaction of aryl halides with amine and nitro precursors^a^

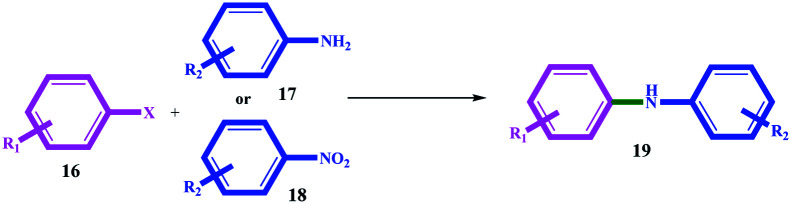								
Entry	R_1_	X	R_2_ (amine or nitro)	Product	Time (h)		Yield^b^ (%)	
					From amine	From nitro^a^^,^^c^	From amine	From nitro
1	H	I	H	19a	3	4.2	90	90
2	4-OH	I	H	19b	5	6.5	90	92
3	4-MeO	I	H	19c	6	7	94	96
4	4-Me	I	H	19d	3.5	4.8	92	96
5	4-CN	I	H	19e	2.2	3.5	96	96
6	H	I	4-OH	19b	2.6	4	95	97
7	H	I	4-MeO	19c	5.5	6.5	94	95
8	H	I	4-CN	19e	2	3.5	98	98
9	H	Br	H	19a	5	6.2	88	90
10	4-OH	Br	H	19b	6.5	7.7	80	80
11	4-MeO	Br	H	19c	7	8	82	85
12	4-Me	Br	H	19d	5	6.5	85	85
13	4-CN	Br	H	19e	3	4	90	86
14	H	Br	4-OH	19b	3	4.5	92	92
15	H	Br	4-MeO	19c	6.5	7.9	88	92
16	H	Br	4-CN	19e	4	5.4	80	85
17	H	Cl	H	19a	8.5	10	75	80
18	4-OH	Cl	H	19b	9	10.5	80	76
19	4-MeO	Cl	H	19c	12	14	66	60
20	4-Me	Cl	H	19d	9	11	75	75
21	4-CN	Cl	H	19e	8.5	10	70	65
22	H	Cl	4-OH	19b	8	9	76	80
23	H	Cl	4-MeO	19c	7	8.5	75	80
24	H	Cl	4-CN	19e	8	9	75	84

a Reaction conditions: amine or nitro (1.0 mmol), aryl halide (1.0 mmol), catalyst 11 (2.0 mg, 0.026 mol% Pd, 0.068 mol% Ni), DMSO_2_ (3.36 g, 35.7 mmol), and 120 °C.

b Isolated yield.

c NaBH_4_ (2.0 mmol).

The results suggest a mechanism based on oxidative-addition and reductive-elimination stages, which is completely consistent with the mechanism proposed in the next section. Coupling reactions with a nitro precursor also proceeded well in the presence of 2 mmol NaBH_4_ in the reaction mixture. The results in Table 4 show that coupling through nitro precursors takes longer, but similar and even higher efficiencies were produced than amines. This can be due to the *in situ* production of amine (*via* the reduction of nitro compounds) and the effect of concentration. In addition, according to the obtained efficiencies, it can be concluded that the reduction of the nitro group to amine (for all the substrates) occurred completely with high selectivity.

### Control experiments

To elucidate the unique catalytic activity of 11, the catalytic activity of different species (homologues) was studied by performing several control reactions for domino oxidation-Suzuki reactions (preparation of 15a) and domino reduction-C–N coupling (preparation of 19a). The results are summarized in Table 5. For a better comparison, the reaction time was considered constant. Catalyst 10 produced very low efficiencies for 15a (45%) during the same time, while the efficiency of 80% was achieved for 19a (Table 5, entry 1). According to these results, it can be deduced that a synergistic and cooperative effect occurs due to the second metal (Ni). In addition, since the reaction requires initial oxidation by alcohol, catalyst 10 showed lower selectivity than 11 (ESI, Table S3†), which can be responsible for the low efficiency observed for the preparation of 15a. Considering this, the presence of the second metal (Ni) controls the selectivity for the oxidation of alcohol to aldehydes. The effect of pyridine groups on the catalyst was also clearly identified by the negligible catalyst activity observed for 13. As shown in Table 5 (entry 2), very low efficiency was observed for 19a, and no efficiency was observed for 15a.

**Table 5:** The control experiments for the domino oxidation Suzuki–Miyaura cross-coupling of benzyl alcohol with phenylboronic acid^a^ and domino reduction C–N cross-coupling of nitrobenzene with iodobenzene^b^

Entry	Catalyst	15a	19a
		Conversion (%)	Conversion (%)
1	Catalyst 10	40	80
2	Polyvinyl chlorophyll-Pd(ii) (13)	N.R.	Trace
3	Chlorophyll b	N.R.	N.R.
4	Fe_3_O_4_	N.R.	N.R.
5	Fe_3_O_4_@SiO_2_	N.R.	N.R.
6	Fe_3_O_4_@SiO_2_–NH_2_	N.R.	N.R.
7	Compound 5	25	65
8^c^	Catalyst 10-poisoned	N.R.	N.R.
9^c^	Catalyst 11-poisoned	5	N.R.
10	Catalyst 11	4^d^	—
11^e^	5/Fe_3_O_4_@SiO_2_–NH_2_/Ni(OAc)_4_·H_2_O	35	30

a Reaction conditions: benzyl alcohol (1.0 mmol), phenylboronic acid (1.0 mmol), catalyst (1.0 mg), DMSO_2_ (3.36 g, 35.7 mmol), 120 °C, O_2_ balloon (∼1.0 atm), 33 min.

b Reaction conditions: nitrobenzene (1.0 mmol), iodobenzene (1.0 mmol), catalyst (2.0 mg), NaBH_4_ (2.0 mmol), DMSO_2_ (3.36 g, 35.7 mmol), 120 °C, 4.2 h.

c Hg(0) was added equal to 320 molar equivalents *vs.* Pd content.

d The preparation of 15a was performed under an N_2_ (sealed) atmosphere.

e 5 (1.0 mg), Fe_3_O_4_@SiO_2_–NH_2_ (2.0 mg), Ni(OAc)_4_·H_2_O (0.1 mg).

These results not only show the vital role of the base in the reactions, but also show that the presence of Ni in the catalyst provides the required electron transitions (between Pd and Ni) for the oxidation-coupling and reduction-coupling reactions, completely in accordance with the proposed mechanism. Chlorophyll, Fe_3_O_4_, Fe_3_O_4_@SiO_2_, and Fe_3_O_4_@SiO_2_–NH_2_ did not produce any observable efficiencies for any of the products 15a and 19a for 32 min and 4.2 respectively (Table 5, entries 3–6).

As shown in entry 7, compound 5 showed less efficiency than 10 (which was only immobilized on the surface of nanoparticles). Considering that 10 and 5 are heterogeneous, this difference can be attributed to the immobilization of the copolymer on the surface of the nanoparticles and the increase in the surface-to-volume ratio, and consequently the increase in catalytic activity.

The catalytic activity of the metal complex moieties as active sites in the catalyst was determined by performing two mercury(0) poisoning control tests for two catalysts, 10 and 11 (Table 5, entries 8 and 9). The reactions with both 15a (from benzyl alcohol) and 19a (from nitrobenzene) stopped, and do detectable efficiency was produced. These results not only indicate the nature and heterogeneity of catalysts 10 and 11 in the reaction medium, but also indicate that the Pd and Ni active sites are responsible for the observed catalytic activity for the oxidation and reduction reactions.

To investigate the reaction mechanism, 15a was prepared using catalyst 11 under a nitrogen atmosphere. For this, the reactor was degassed for 5 min and then the catalyst was added to the reaction mixture simultaneously. After 33 minutes, only 4% conversion was observed (Table 5, entry 10). This result well demonstrates the effect of molecular oxygen (as a source of oxygen) and the subsequent coupling reaction through the aldehyde intermediate. Finally, the catalytic activity of the single components of catalyst 11 was studied in a control experiment. For this, a mixture of 5/Fe_3_O_4_@SiO_2_–NH_2_/Ni(OAc)_4_·H_2_O was evaluated for the preparation of 15a and 19a (Table 5, entry 11). The efficiency was found to be 35% for 15a, which was higher than that for compound 5 (Entry 7). This slight increase can be attributed to the catalytic effect of nickel salt in the reaction mixture. However, the efficiency of the catalytic mixture for the preparation of 19a (30%) was less than that of compound 5. The results show the effect of Ni metal coordination on the catalytic structure for its reducing activity. In addition, these results indicate the unique catalytic activity of 11 relative to its components alone, suggesting a consistent correlation between the various components with a potential synergistic effect.

### Chemoselectivity

Chemoselectivity studies give useful information regarding the selectivity and activity of a catalyst. Five different combinations of alcohols and amines were selected for the domino oxidation-C–C coupling and domino-reduction C–N coupling reactions. Table 6 shows the corresponding results. The combination of phenyl boronic acid, 4-NO_2_-benzyl alcohol, and benzaldehyde, gave 15a selectively; *i.e.* decarbonylative coupling with benzaldehyde instead domino oxidation-coupling (Table 6, entry 1).

**Table 6:** Chemoselectivity behavior of catalyst 11 over the domino oxidation-C–C coupling and domino-reduction C–N coupling reactions in various combinations^a^^,^^b^

Entry	Combination	Selectivity^c^ (%)							Time (h)	Conversion^c^^,^^d^ (%)
		15a	15f	BB	OxP	19a	19e	RP		
1	PB + NBA + B	92	0	—	7	—	—	—	30	79
2	PB + NBA + I	34	0	—	66	—	—	—	1.5	25
3	PB + NBA + BA	—	99	0	0	—	—	—	30	98
4	I + NN + An	—	—	—	—	96	4	0	3	94
5	I + NN + BAm	—	—	—	—	99	0	0	3	94

a Definition: BB: butyl benzene; OxP: oxidation products; RP: reduction products; PB: phenylboronic acid; NBA: 4-NO_2_-benzyl alcohol; B: benzaldehyde; I: iodobenzene; BA: *n*-butanol; NN: 4-CN-nitrobenzene; An: aniline; and BAm: *n*-butyl amine.

b Reaction conditions: for each combination, 1.0 mmol of each reactant was used. For entries 1–3: see Table 3 footnote (O_2_ balloon ∼1.0 atm). For entries 4 and 5 see Table 4 footnote (NaBH_4_: 2.0 mmol).

c GC analysis.

d For major coupling product.

The catalyst did not show satisfactory selectivity in the presence of iodobenzene, wherein the C–C coupling reaction was performed at 1.5 h with 25% conversion for 15a (entry 2). However, 66% selectivity was obtained for 4-NO_2_-benzaldehyde for this combination. On the other hand, 4-NO_2_-benzyl alcohol selectively coupled with phenylboronic acid in the presence of butyraldehyde and the oxidation product was also found, reflecting the selectivity of the catalyst towards aromatic aldehyde (entry 3). Two combinations were also performed for the domino reduction-C–N coupling, where in first, the combination of iodobenzene, 4-CN-nitrobenzene, and aniline, selectively gave 19a with 94% conversion (Table 6, entry 4). Similarly, the aromatic 4-CN-nitrobenzene was selectively coupled without interference from the aliphatic amine present in the mixture (Table 6, entry 5).

In conclusion, the cross-coupling with aldehyde and amine is superior to the domino oxidation- and reduction coupling, and the coupling of aromatic substrates can be selectively coupled compared to the aliphatic type.

### Mechanism studies

**Scheme 5:**
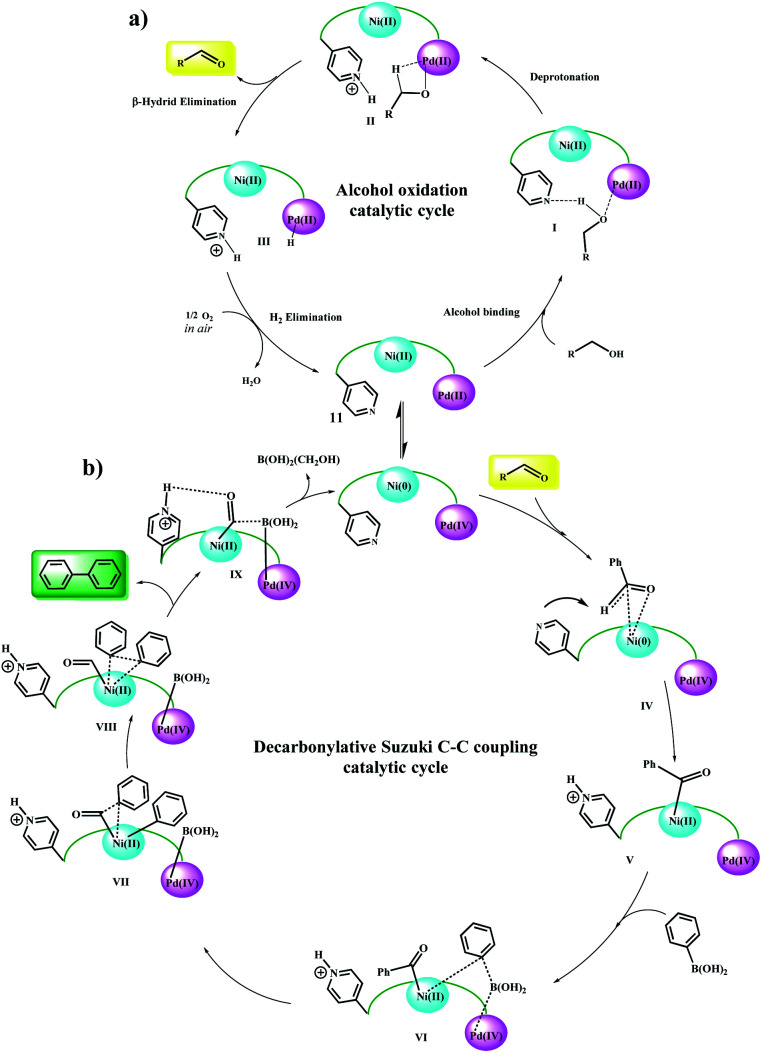
Plausible reaction mechanism for (a) Ni/Pd-catalyzed oxidation of alcohol and (b) domino oxidation Suzuki–Miyaura cross-coupling of alcohols.

According to the mechanism reported by Guo *et al.*^20^ for the nickel-catalyzed decarbonylative arylation reaction of aldehydes with boronic esters, and based on our observations, a general reaction mechanism was proposed for the Fe_3_O_4_@SiO_2_/(Py)-*copolymer*-(chlorophyll b) Ni/Pd-catalyzed C–C cross-coupling reactions. Previously, alcohol oxidation to the corresponding carbonyl group could be performed using any Pd or Ni active site. However, it is not possible to determine with certainty which metal exhibits the desired catalytic activity, and it may be done either alone or in collaboration with a synergistic effect, as reported by Nasseri *et al.* for the Co–Cu bimetallic catalytic system for coupling reactions.^11^ Therefore, the oxidation mechanism of alcohols is selectively sketched for Pd. According to this proposed mechanism (Scheme 5a), the alcohol is initially approached by the oxygen groups to the Pd sites and a possible hydrogen bond forms between the Py and oxygen groups. In the next step, with the abstraction of protons by the Py groups, the alcohol is coordinated to the Pd centers. β-Hydride elimination leads to the carbonyl product. The protons adsorbed by the Pd and Py centers are received by molecular oxygen and converted to water, and the catalyst returns to the cycle. It has been shown that the simultaneous presence of two coordinated metals in a catalytic structure allows the transfer of electrons between centers.^22–26^

**Scheme 6:**
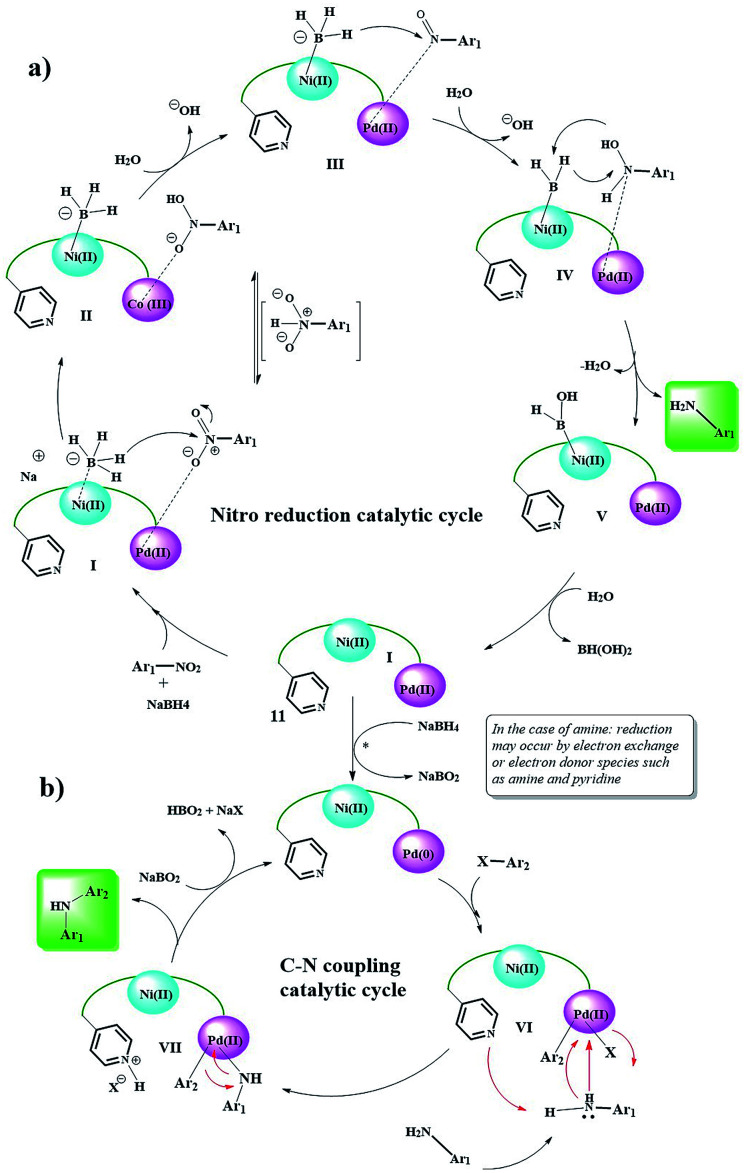
Plausible reaction mechanism for the Ni/Pd-catalyzed reduction of nitro to amine and domino reduction C–N cross-coupling of nitro compounds with aryl halides.

Here, again, the electron exchange between the Pd and Ni centers creates the 0 and IV oxidation states for Ni and Pd, respectively, and prepares the catalyst for the coupling reaction. The proposed mechanism is completely consistent with these results. The aldehyde group approaches the Ni(0) centers through the C=O bond and acylates it, and the corresponding proton is taken up by the Py group (V).^20^ Then, phenylboronic acid approaches to catalyst surface with the help of the Pd centers and through intermediate VI, leading to the phenyl coordination and formation of Pd(iv)–B(OH)_2_ groups. The coupling product is formed after several intermediates and the catalyst returns to the cycle by removing B(OH)_2_(CH_2_OH).^27–29^ Control experiments (Table 5) were performed to help understand this mechanism. Elimination of any of the catalytic centers (Table 5, entries 1, 2, 8 and 9) and removal of oxygen (Table 5, entry 10) caused the catalytic activity to stop. This indicates that all parts of the catalyst, including the Pd, Ni centers and Py groups are involved in the reaction and suggests a synergistic effect according to the mechanism shown in the scheme. Subsequently, a possible mechanism for the reduction of nitro compounds to amine and subsequent C–N coupling was proposed in accordance with the observations obtained from the control experiments and the reported mechanisms (Scheme 6).^30,31^

According to this mechanism, NaBH_4_ and the nitro groups on the surface of the catalyst are coordinated/linked through the Ni and Pd centers, respectively, and in several steps of hydride transfer from the BH_4_ groups to nitro (intermediates I, II, III, and IV),^30–32^ are reduced to an arylamine. Due to the presence of NaBH_4_ groups in the medium, the reduction of Pd(ii) to Pd(0) seems probable (in the case of direct the coupling of arylamines with aryl halides, this process can also be performed by amine groups^33,34^). Then, by an oxidative-addition reaction, aryl halide is added to Pd and the Pd centers are oxidized to +2. In the next step, the amine group is attached to the Pd centers *via* a nitrogen electron pair, and the amino group proton is taken by the Py group present in the catalyst.

Finally, the C–N coupling product is formed, and the Pd centers are regenerated to the zero oxidation state. Finally, the protonated Py groups are treated with NaBO_2_ (formed in the previous step), and the catalyst is returned to the cycle.

In addition, to confirm the absence of radical species in the reaction mixture, and subsequently confirm the non-radical mechanism in the coupling reactions, the preparation of 15a and 19a in the presence of hydroquinone, as an electron capture agent, was performed from the beginning. Fig. S10† shows the effect of the presence of hydroquinone in comparison with the normal kinetics for both reactions. The reaction rate was monitored at different times. According to Fig. S10a and b,† hydroquinone had no effect on the efficiency and subsequent kinetics of the reaction, which reflects the absence of any radical (and radical mechanism) in both reactions, in full agreement with the proposed mechanism.

Due to the presence of multiple catalytic centers in the Ni/Pd catalyst, the recoverability, metal leaching, and heterogeneity of the catalyst were studied. For this purpose, the catalyst recovery was performed in two coupling reactions of domino C–C coupling of benzyl alcohol with iodobenzene in the presence of molecular O_2_ and also domino C–N coupling of nitrobenzene with iodobenzene in the presence of NaBH_4_. Both reactions were performed under the optimal conditions for the appropriate times according to Tables 3 and 4.

**Fig. 1:**
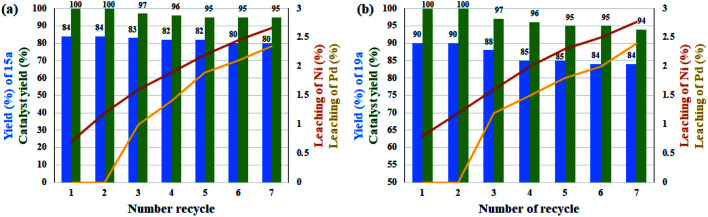
Recyclability and leaching studies of the catalyst over the reaction of (a) benzyl alcohol with phenylboronic acid and (b) nitrobenzene with iodobenzene.

Leaching experiments for the domino preparation of 15a and 19a in each cycle from the residual mixture were studied by ICP analysis for both Ni and Pd metals. The results are shown as curves in Fig. 1. The amount of leaching in each cycle was very small and at the end of the seventh cycle. A total of 2.35% and 2.77% were observed for Pd and 2.66% and 2.4% for Ni for products 15a and 19a, respectively. This slight leaching caused a slight decrease in catalytic activity for the preparation of compounds 15a and 19a, and thus the efficiency declined to 95% and 94%, respectively, after 7 consecutive runs. Interestingly, the drop rate was zero for Pd up to the third cycle for both reactions. This stability can be directly attributed to the better coordination of Pd ions in the rigid structure of porphyrins than the salen ligand for Ni ions. In addition, the results showed the good stability for the catalyst in successive cycles (Fig. 1). The heterogeneous performance of the catalyst in the reaction medium was shown previously by mercury poisoning experiments (Table 5, entries 8 and 9) for catalysts 10 and 11 for the Suzuki–Miyaura domino oxidation cross-coupling of benzyl alcohol with phenylboronic acid and domino reduction C–N cross-coupling of nitrobenzene with iodobenzene, respectively. In addition, the hot filtration test for catalyst 11 in the reaction for the Suzuki–Miyaura domino oxidation cross-coupling of benzyl alcohol with phenylboronic acid was studied. For this, the catalyst was magnetically filtered from the reaction medium after 15 h. The reaction progress was 48% at this time, after which the reaction proceeded for another 15 h in the absence of the catalyst. Subsequently, the efficiency reached to 50% (with negligence). Therefore, the results once again confirmed the heterogeneity of the catalyst and showed that the amount of metal leaching in the reaction medium is very small and unlikely.

**Fig. 2:**
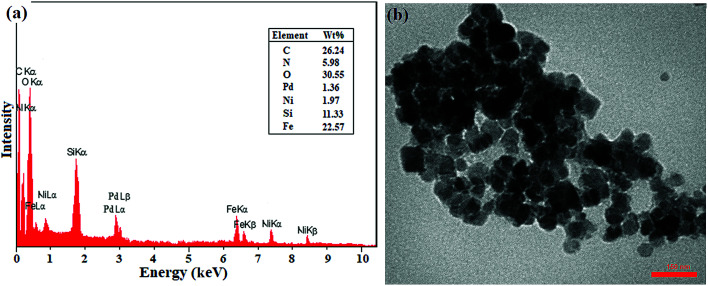
(a) EDX spectrum and (b) TEM image of the recovered catalyst after the 7^th^ recycling of the domino oxidation-coupling reaction of benzyl alcohol with phenylboronic acid.

In addition, the recovered catalyst was characterized by TEM and EDX analyses after the end of the seventh cycle in the domino C–C coupling of benzyl alcohol with phenylboronic acid. As shown in Fig. 2, the EDX analysis showed the presence of all elements at exactly the same percentages as that of the freshly prepared catalyst. However, the decrease in the percentages of Ni and Pd can be directly attributed to the metal leaching, which is completely consistent with the results of the leaching study (Fig. 2). The TEM image also showed the same morphology and particle size as that of the freshly prepared catalyst. Thus, the results showed the good stability of the catalyst in terms of structure and morphology during successive cycles.

## Conclusion

Herein, a stable multifunctional catalyst bearing a hetero-magnetic solid support, base functionality, and coordinated metal sites (including Ni and Pd) with redox catalytic property was successfully synthesized as a single catalytic system for the domino oxidation-C–C and reduction-C–N cross-coupling reactions. The catalyst was characterized using various analytical and spectroscopic techniques including GPC, FTIR, UV-Vis, EDX, FE-SEM, TEM, NMR, XPS, DLS, VSM, DET, and ICP analyses. Domino C–C coupling of alcohols with aryl halides and domino C–N cross-coupling of nitro compounds with aryl halides were performed in the presence of the Ni/Pd catalyst, and moderate efficiency and high selectivity were achieved toward a variety of substrates. The cooperative performance of the catalyst between the Ni sites, Pd sites, and pyridine moieties was responsible for this excellent activity, which was confirmed by various control experiments. The results also confirmed the synergistic effect of Ni and Pd sites for domino oxidation–reduction reactions, which promoted the efficiency and selectivity. The chemoselectivity studies demonstrated that the cross-coupling with aldehyde and amine is superior to the domino oxidation- or reduction coupling, and the coupling of aromatic substrates could be selectively coupled compared to the aliphatic type. The catalyst could be recycled for at least 7 consecutive runs with an insignificant loss in its reactivity for both the C–C and C–N coupling reactions.

## Experimental

### Materials and instruments

All chemicals were obtained from Sigma and Fluka and used without further purification. All solvents were distilled and dried before use. All other reagents were of analytical grade. Dimethyl sulfone (DMSO_2_) crystals were prepared according to a previously reported procedure^11^ and used as the solvent in the reactions. Reaction progress was monitored by thin layer chromatography (TLC) on silica gel (Polygram SILG/UV 254 plates) or gas chromatography (GC) using a Shimadzu-14B gas chromatography equipped with an HP-1 capillary column and N_2_ as the carrier gas and anisole as an internal standard. FTIR spectra were recorded using a JASCO FT/IR 4600 spectrophotometer using KBr discs. ^1^H NMR (250 MHz) and ^13^CNMR (62.9 MHz) spectra were recorded on a Bruker Avance DPX-250 spectrometer in deuterated solvents, CDCl_3_ and DMSO-*d*_6_, with TMS as an internal standard. X-ray diffraction (XRD) analyses were performed with a Bruker D8/Advance powder X-ray diffractometer. The cell temperature was maintained at 25.0 ± 0.1 °C using a HAAKE D8 recirculating bath. Elemental analyses were performed on a PerkinElmer-2004 instrument. Field emission scanning electron microscopy (FE-SEM) images were obtained on a HITACHI S-4160 and TESCAN MIRA3 instrument. Elemental analysis (EDX) spectroscopy was performed using a field emission scanning electron microscope, FESEM, JEOL 7600F, equipped with an energy dispersive X-ray spectrometer from Oxford Instruments. Transmission electron microscopy (TEM) was performed on a Philips EM208 microscope operated at 100 kV. The size distribution of the nanoparticles was measured by dynamic light scattering (DLS) on a HORIBA-LB550 apparatus. The magnetic behavior of the samples was measured on a Lake Shore vibrating sample magnetometer (VSM) at room temperature. ICP experiments were performed using a VARIAN VISTA-PRO CCD simultaneous ICP-OES instrument. The average molecular weight of the samples was measured by the gel permeation chromatography (GPC) using a Knauer Advanced Scientific Instrument, Germany, with an RI detector (Smartline 2300) PL gel 10 μm, 10 × 10^3^ Å column with 20 μL injected volume. Monodispersed poly(methyl methacrylate), PMMA, standards were used for calibration. The average molar mass was determined using the Millennium 2010 software. The surface area, pore volume, and pore diameter of the obtained NPs were measured by N_2_ physisorption at −196 °C with a surface area and pore size analyzer (Micromeritics ASAP 2000 instrument) using the BET method. X-ray photoelectron spectroscopy (XPS) investigations were conducted on an XR3E2 (VG Microtech) twin anode X-ray source with Al-Kα = 1486.6 eV.

### Demetalation of chlorophyll b (2)

Chlorophyll b was extracted from *Heliotropium europaeum* plant by simply extracting 1.0 g of dried plant powder in 30 mL of 80% acetone, and then filtered.^35^ The chlorophyll b was purified by silica gel column chromatography.^36–38^ The following mobile phases were applied with the order of: 60% *n*-hexane, 16% cyclohexane, 10% ethyl acetate, 10% acetone, and 4% methanol. The elution order using this elution solvent system was β-carotene, chlorophyll b, and xanthophyll (from top to bottom). Commercial purified samples of β-carotene and chlorophyll b were used as controls. For the demetalation of chlorophyll b, an acetone solution of the chlorophyll (5.0 mM) was prepared according to previous works. Then, 1.0 mL of 0.5 M HCl was added to 3.0 mL of acetone solution of chlorophyll in a round-bottom flask. The reaction was stirred for 2 h at room temperature. Then, the mixture was extracted by *n*-BuOH (3 × 10 mL). Finally, the solvent was removed under reduced pressure and the resulting product was completely dried in a vacuum oven at 50 °C for 12 h (Scheme 3).

### Preparation of chlorophyll-allyl (3)

The extracted chlorophyll b (1.0 g, 1.0 mmol) was dissolved in 20.0 mL of absolute methanol. A methanolic solution of allylamine (3.0 mmol in 20 mL methanol) was added dropwise for 30 min to the chlorophyll solution. The color immediately changed to green-yellow and the mixture was stirred at room temperature for 2 h. Subsequently, the pale green solid was filtered, washed with deionized water and dried at room temperature in a vacuum desiccator.

### Preparation of (Py)-*co*-(chlorophyll b) polymer (4)

Copolymerization/grafting 4-vinylpyridine to chlorophyll-allyl was performed according to a previously reported procedure.^39^ Typically, 4-vinylpyridine (0.15 g) and chlorophyll-allyl (3) were added to a dried round-bottom flask. The flask was nitrogen-purged for two minutes, and then 6.0 mL dioxane, 6.0 mg AIBN was added to the flask. The system was sealed and equipped with an N_2_ inlet and then immersed in an oil bath. The mixture was stirred at 85 °C for 24 h. Then, the solution was allowed to cool to room temperature and added to excess MeOH as a precipitating solvent in one step. Copolymer 4 was obtained after the removal of the solvent under reduced pressure. The product was purified by treatment with diethyl ether (25 mL), and then dried under vacuum at room temperature for a day (Scheme 3).

### Preparation of (Py)-*grafted*-(chlorophyll b)-Pd(ii) complex (5)

Coordination of Pd ions to 4 (as a ligand) was performed as follows: copolymer 4 (0.5 g) was added to 25 mL EtOH at 50 °C, and then PdCl_2_·2H_2_O (0.04 g, 0.2 mmol) was added to the mixture. The mixture was stirred for 2 h, and then filtered, washed with dry toluene (2 × 10 mL), and dried in an oven (60 °C).

### Self-catalytic cross-coupling of (Py)-*copolymer*-(chlorophyll b)-Pd(ii) complex with 4-iodo salicylaldehyde (6)

The self-catalytic base-free coupling of (Py)-*grafted*-(chlorophyll b)-Pd(ii) complex with 4-iodo salicylaldehyde was performed in DMF (10 mL) in the absence of any base. Compound 5 (0.5 g) was added to 10 mL DMF under reflux conditions. Then, 4-iodo salicylaldehyde (20.0 mmol) dissolved in 10 mL DMSO was added to the above mixture. Then, the mixture was stirred for 24 h. Subsequently, the mixture was poured in excess 50 mL cold EtOH. The precipitate was filtered, washed with acetone and DEE (each 2 × 10 mL). The product was dried in an oven and isolated at a refrigerator.

### Preparation of Fe_3_O_4_@SiO_2_–NH_2_ NPs (9)

Fe_3_O_4_@SiO_2_–NH_2_ (9) was prepared through three steps according to a procedure described elsewhere.^40^

### Immobilization of (Py)-*copolymer*-(chlorophyll b)-Pd(ii)-epoxide on Fe_3_O_4_@SiO_2_–NH_2_ (10)

(Py)-*copolymer*-(Chlorophyll b)-Pd(ii) epoxide (6) was immobilized on Fe_3_O_4_@SiO_2_–NH_2_ magnetic NPs in one step. Fe_3_O_4_@SiO_2_–NH_2_ (1.0 g) was dispersed ultrasonically in 10 mL DMSO for 20 min at room temperature. Then, 0.5 g (Py)-*copolymer*-(chlorophyll b)-Pd(ii) (6) in 15 mL DMSO was added dropwise to the mixture under ultrasonic conditions. The addition took 30 min, and then the mixture was refluxed for 24 h. The resulting product was collected by an external magnetic field, washed with deionized water and EtOH (each 2× 15 mL), an then dried in a vacuum oven (Scheme 3).

### Preparation of Fe_3_O_4_@SiO_2_/(Py)-*copolymer*-(chlorophyll b)-Pd(ii) (11) by coordination of Ni ions to 10

For the coordination of Ni ions to the catalyst framework, 0.5 of 10 was dispersed in 25 mL absolute EtOH at room temperature for 20 min. Then Ni(OAc)_2_·4H_2_O (0.05 g, 0.2 mmol) was added to the mixture. The mixture was refluxed with stirring for 4 h. Then, the resulting product was filtered using an external magnetic field and washed with deionized water and ethanol several times to remove of any metal salt, and then dried in an oven.

### Acid titration test for the determination of the content of Py monomer in the catalyst

An acid titration assay was used to prove and quantify the grafting of the Py moieties in copolymer 4. In this test, 1.2 g of 4 was added to 50 mL of distilled water and sonicated for 10 min. The copolymer was titrated with 0.2 M acetic acid in the presence of phenolphthalein indicator under ultrasonic conditions. About 9.5 mL acetic acid was consumed at the end point. Simultaneously, a blank was also titrated, and the total volume of acetic acid was recorded.

### General procedure for Ni/Pd-catalyzed domino oxidation Suzuki–Miyaura cross-coupling from alcohols

Generally, a 10 mL round-bottom flask equipped with a magnetic stirrer bar and condenser was charged with alcohol (1.0 mmol), phenylboronic acid (1.0 mmol), catalyst 11 (0.013 mol% Pd), and DMSO_2_ (3.36 g, 35.7 mmol). An O_2_ balloon (∼1.0 atm) was installed and the mixture temperature was adjusted to 120 °C. The reaction progress was monitored by TLC. Upon completion of the reaction, the catalyst was removed magnetically after cooling the mixture to room temperature, washed with deionized water and EtOH (each 3 × 5.0 mL), and then dried and stored for the next run. For the extraction of the product, EtOAc (5.0 mL) and H_2_O (5.0 mL) were added to the residue. The resulting aqueous phase was further extracted in EtOAc (2 × 5.0 mL). The organic layers were combined and dried over Na_2_SO_4_, and EtOAc was removed under reduced pressure. The pure coupling product was obtained by flash chromatography of the crude product.

### General procedure for Ni/Pd-catalyzed reduction of nitro to amine and domino reduction C–N cross-coupling of nitro compounds with aryl halides

In a 10 mL round-bottom flask, nitroarene (1.0 mmol), aryl halide (1.0 mmol, in the case of C–N coupling), catalyst 11 (2.0 mg, 0.026 mol% Pd, 0.068 mol% Ni), DMSO_2_ (3.36 g, 35.7 mmol), and NaBH_4_ (2.0 mmol) were mixed and the reaction temperature was adjusted to 120 °C. The reaction was stirred at constant temperature and the progress was monitored by TLC based on aryl halide consumption. The catalyst separation and isolation of the desired C–N coupling product was the same as the aforementioned procedure for the domino oxidation C–C coupling.

## Conflicts of interest

There are no conflicts to declare.

## Supplementary Material

RA-010-D0RA08344E-s001
