# Supplementary material for: Ni/Pd-catalyzed Suzuki–Miyaura cross-coupling of alcohols and aldehydes and C–N cross-coupling of nitro and amines via domino redox reactions: base-free, hydride acceptor-free
Source: RSC Adv. 2020 Dec 10;10(72):43962–74. doi: 10.1039/d0ra08344e (PMC9058410; doi:10.1039/d0ra08344e)
Supplement: RA-010-D0RA08344E-s001 [file RA-010-D0RA08344E-s001.pdf]

Electronic Supplementary Material (ESI) for RSC Advances.
This journal is © The Royal Society of Chemistry 2020

Supplementary Information

Ni/Pd catalyzed Suzuki-Miyaura cross coupling of alcohols and aldehydes and C-N cross-couplings of nitro and amines *via* a domino redox reactions: Base-free, hydride acceptor-free

Milad Kazemnejadi*[a], Rebin Omer Ahmed, Boshra Mahmoudi

[a] *Department of Chemistry, College of Science, Shiraz University, Shiraz 7194684795, Iran.*
[b] *Anwar Shekha Medical City, Sulaymaniyah, Iraq, Zip code 46024.*
[c] *Research Center, Sulaimani Polytechnic University, Sulaimani, Iraq.*

General procedure for the preparation of Polyvinyl chlorophyll-Pd complex (13)

Radical polymerization of chlorophyll-allyl was performed according to a previously reported procedure [1]. Typically, chlorophyll-allyl (0.15 g) was added to a dried bottom flask. The flask was nitrogen-purged for 2.0 min, then 6.0 mL dioxane, 6.0 mg AIBN (as an initiator) was added to the flask. The system was sealed and equipped with a N_2 inlet and then immersed in an oil bath. The mixture was stirred at 85 °C for 24 h. Then, the solution was allowed to cool to room temperature and added to excess MeOH as a precipitating solvent in one step. The product was obtained after removal of solvents under reduced pressure. The product (polyvinyl chlorophyll) was purified with treatment with diethyl ether (25 mL), then it was dried under vacuum at room temperature for a day (**12**). Average molecular weight= 11440, polydispersity index = 1.212, DP according to GPC analysis= 10.

Coordination of Pd ions to polyvinyl chlorophyll (as a ligand) was performed as follows: Polyvinyl chlorophyll (0.5 g) was added to 25 mL EtOH at 50 °C, then $Pd(OAc)_2$ (0.045 g, 0.2 mmol) was added to the mixture. The mixture was stirred for 2 h, then it was filtered, washed with dry toluene (2 × 10 mL), and dried into oven (60 °C). ICP = 3.25 %wt Pd.

Conversion and selectivity for carbonyl and amine were calculated using the following formula [2]:

$$\text{Conversion (mol\%)}=\frac{\text{(initial mol\%)-(final mol\%)}}{\text{initial mol\%}}\times 100 \qquad (1)$$

$$\text{Selectivity}=\frac{\text{GC peak area of desired product}}{\text{GC peak area of all products}}\times 100 \qquad (2)$$

General procedure for Ni/Pd and Pd catalyzed oxidation of alcohol

The catalytic activity of **10** and **11** was also evaluated over the oxidation of alcohols. A 10 mL round bottom flask equipped with a magnetic stirrer bar and condenser, was charged with alcohol (1.0 mmol), catalyst (**10** or **11**, 0.013 mol%Pd), and $DMSO_2$ (3.36 g, 35.7 mmol). An O_2 balloon (~ 1.0 atm.) was installed and the mixture temperature was adjusted to 120 °C. The reaction progress was monitored by GC instrument. Upon reaction completion, the catalyst (**10** or **11**) was removed magnetically after cooling the mixture to room temperature, washed with deionized water and then EtOH (each 3×5.0 mL), then dried and stored for the next run.

Catalyst characterization

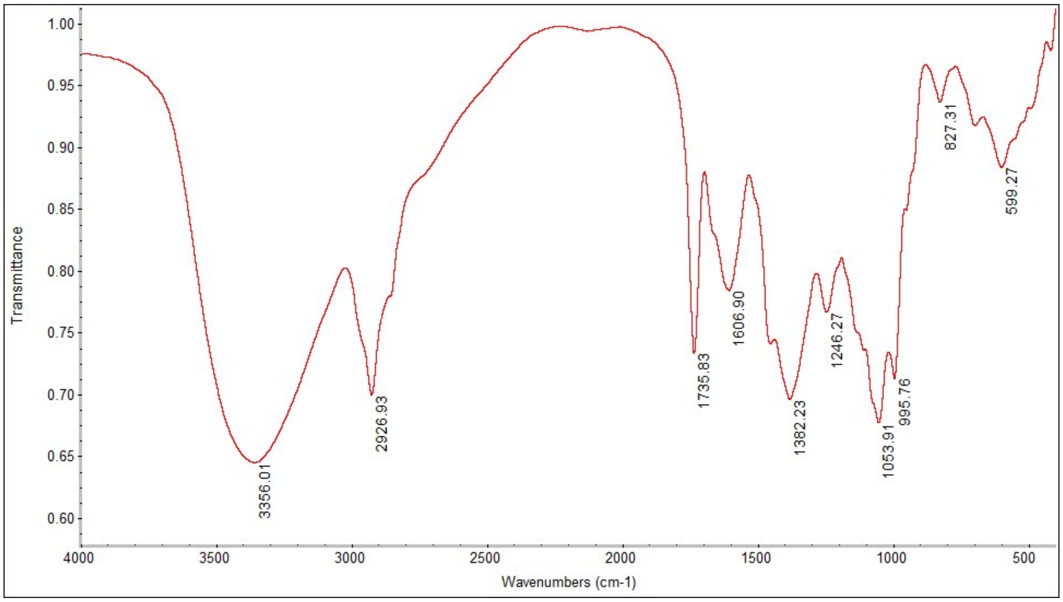

Fig. S1 FTIR spectrum of chlorophyll *b* extracted from *heliotropium europaeum*

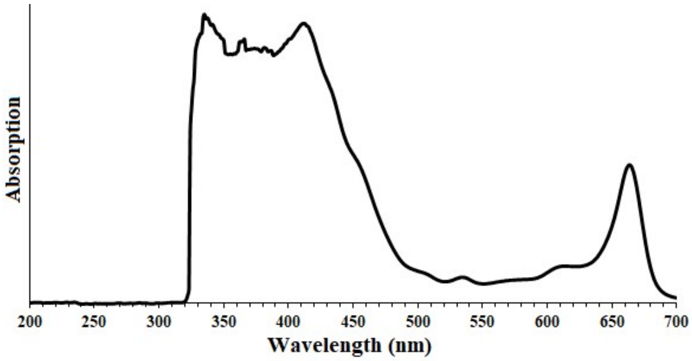

Fig. S2 UV-Vis spectrum of chlorophyll *b* extracted from *heliotropium europaeum* in EtOH

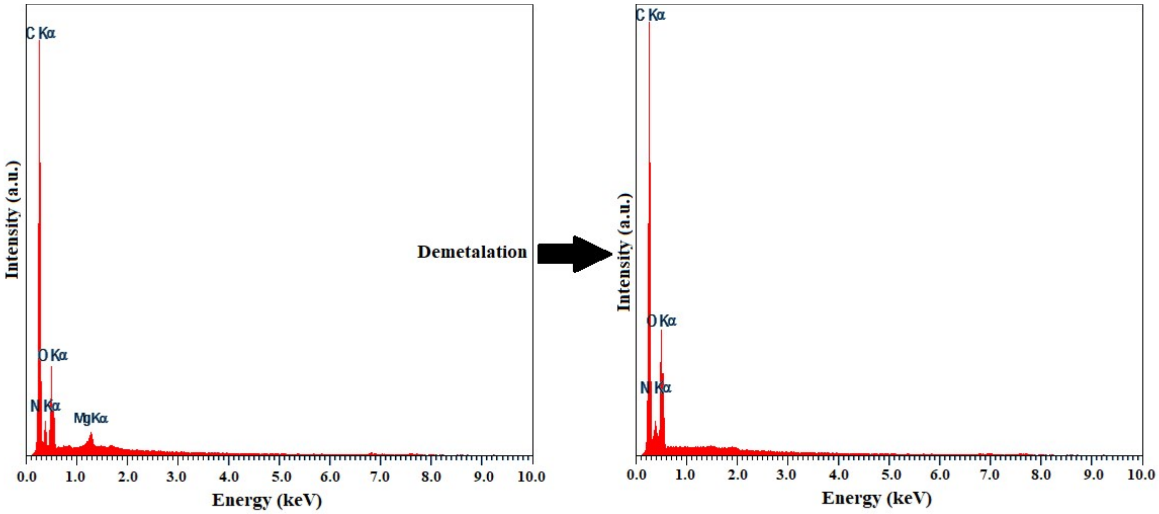

Fig. S3. EDX analysis of (a) chlorophyll *b* (**1**) and (b) demetalated chlorophyll *b* (**2**)

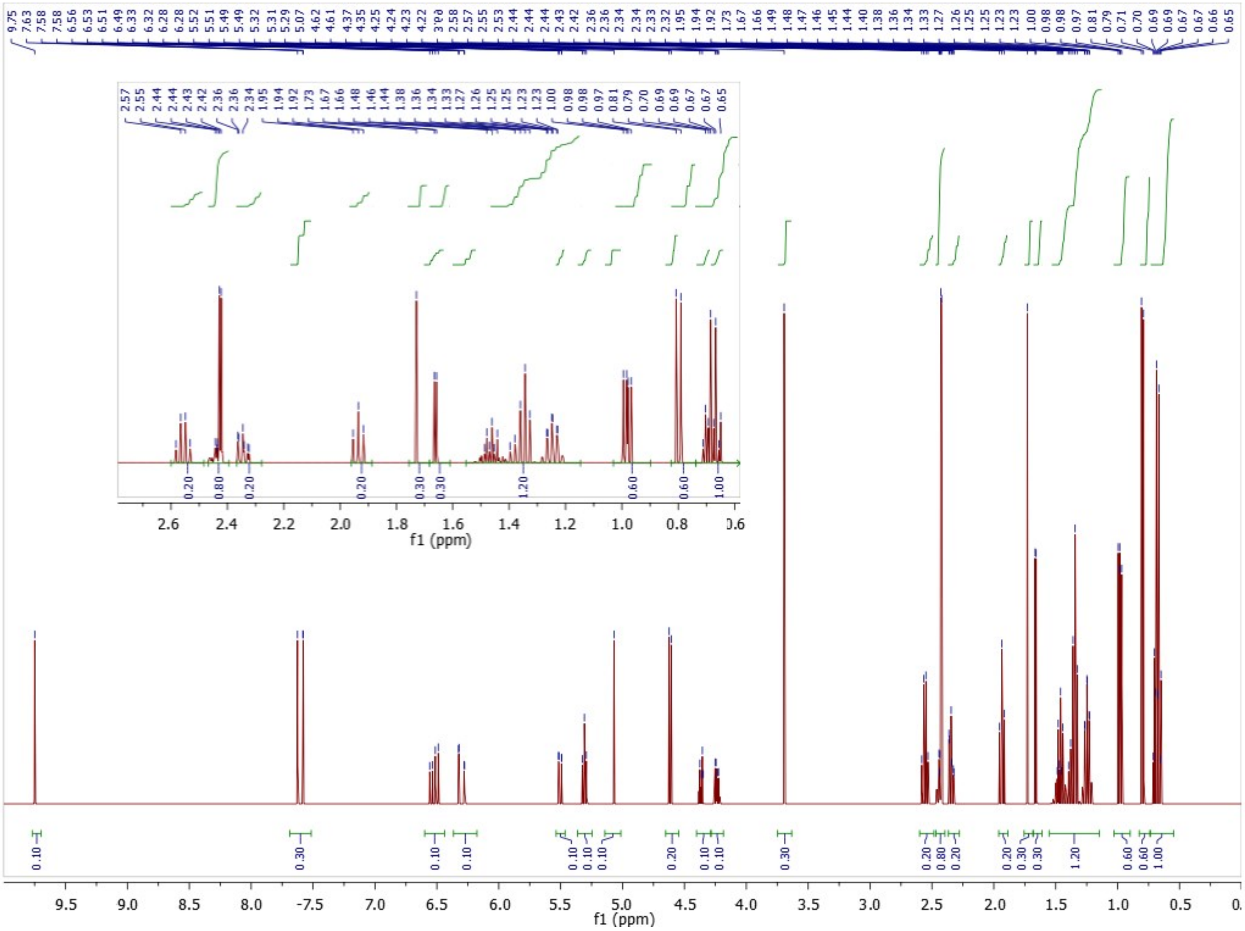

Fig. S4 ^{1}HNMR spectrum of the demetalated chlorophyll b (**2**) in D_2O (400 MHz)

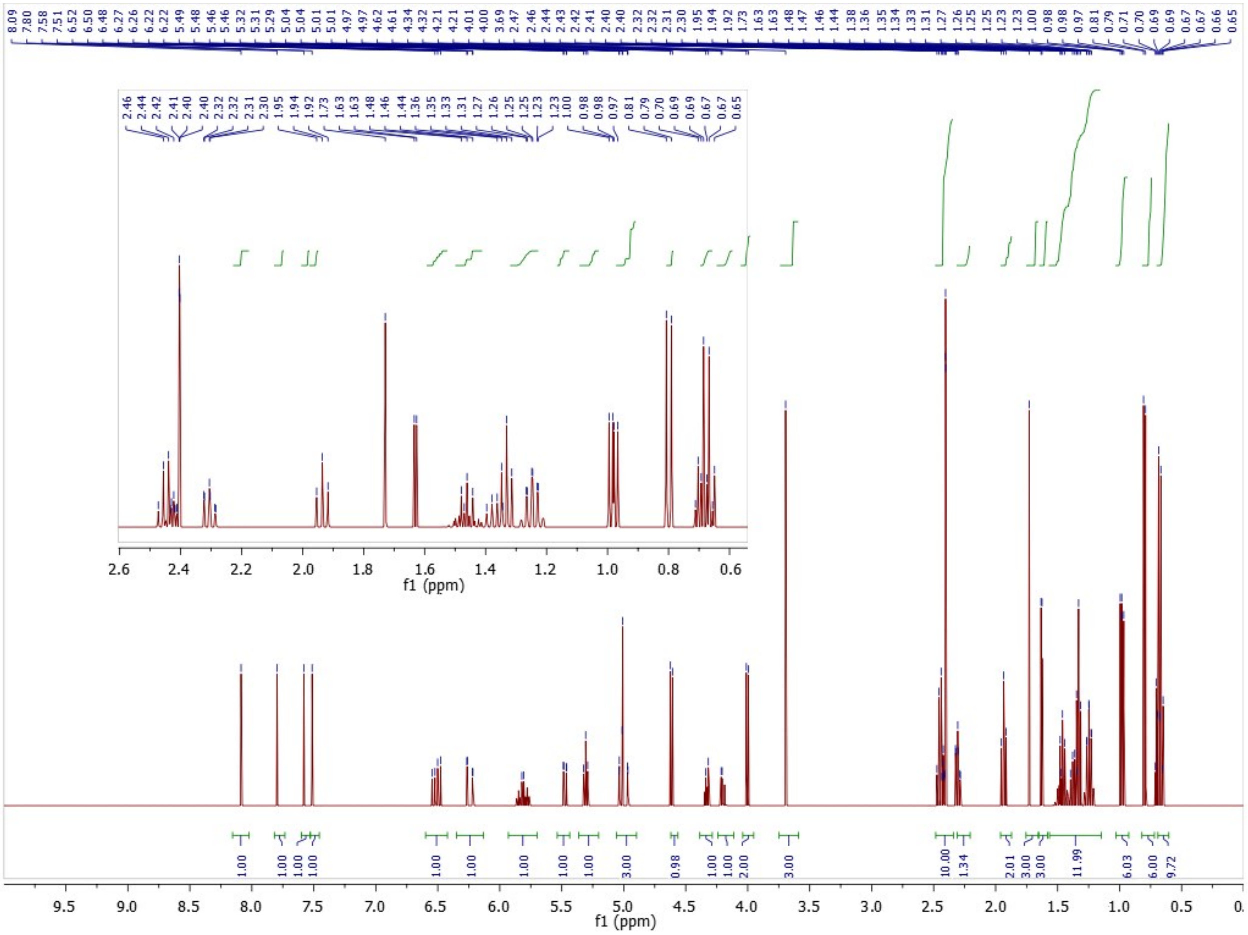

Fig. S5 ^{1}HNMR spectrum of the chlorophyll-allyl (**3**) in D_2O (400 MHz)

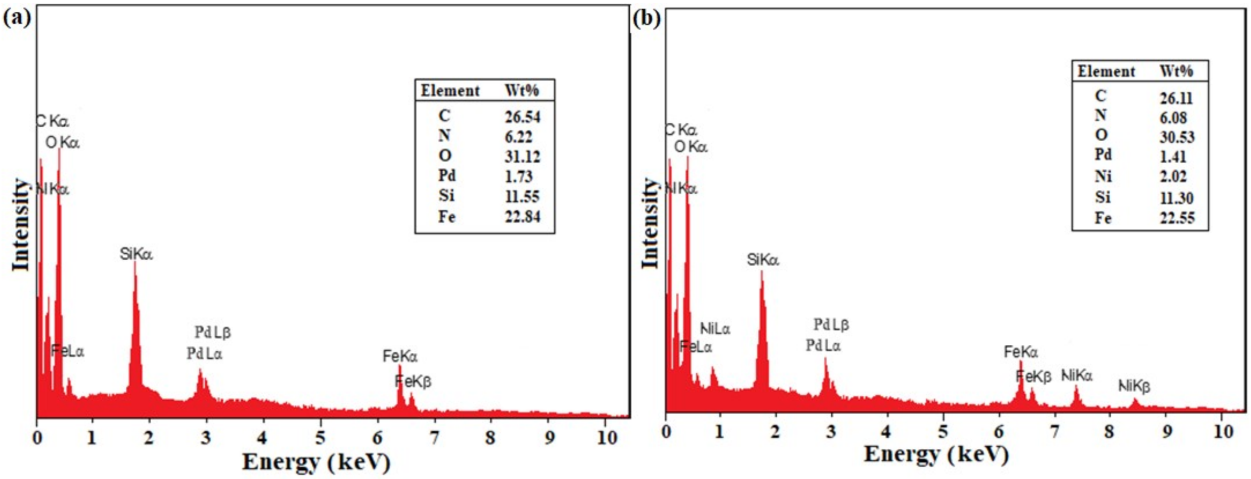

Fig. S6 EDX spectrum of (a) **10** and (b) **11**. The inset tables show the analysis average of five points.

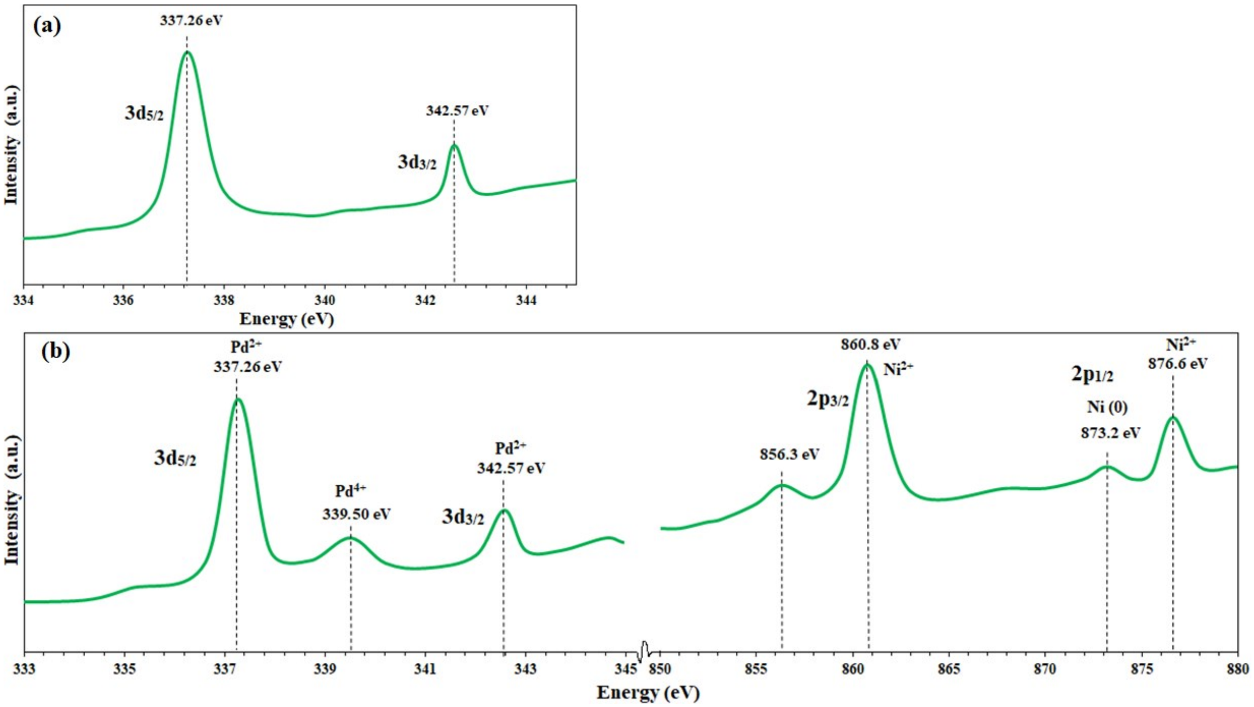

Fig. S7 High resolution XPS analyses (energy corrected) of (a) Pd 3d of **10** and (b) Pd 3d and Ni 2p of **11**.

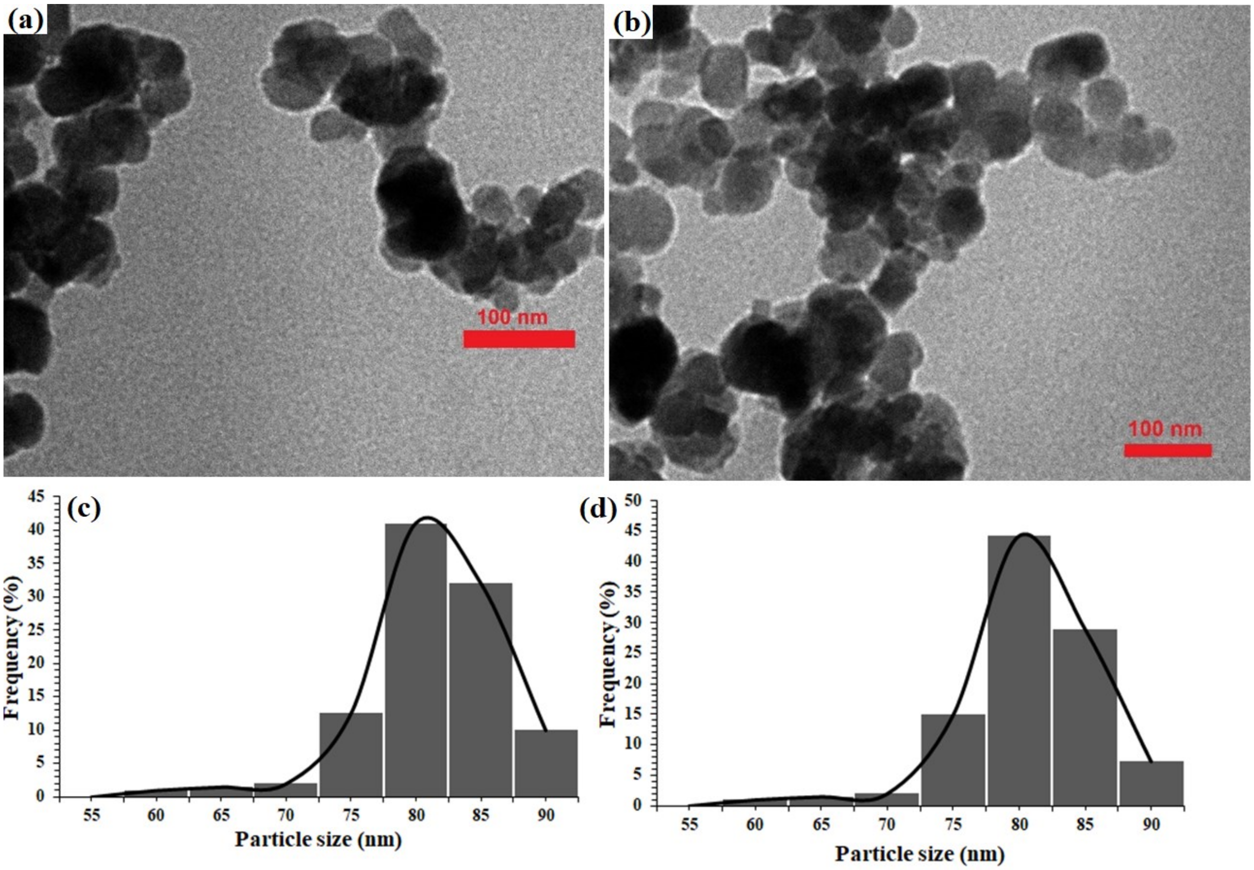

Fig. S8 (a) TEM and (c) DLS analyses of **10**. (b) TEM and (d) DLS analyses of **11**.

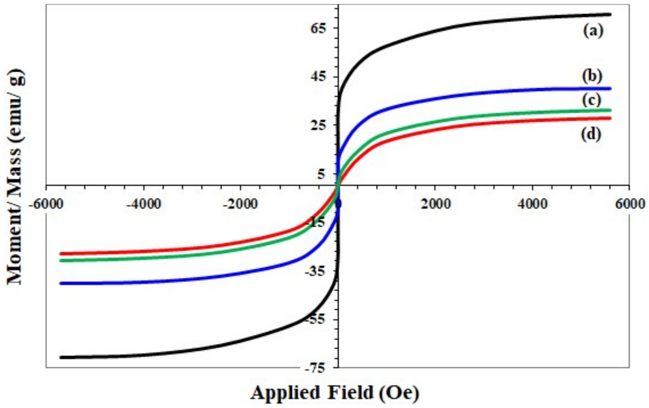

Fig. S9 VSM curves of (a) Fe_3O_4, (b) $Fe_3O_4@SiO_2$, (c) **10**, and (d) **11**.

Table S1 Surface characteristics of Fe_3O_4, $Fe_3O_4@SiO_2$ $Fe_3O_4@SiO_2$-NH_2, **10**, and **11**

Entry	Sample	Specific surface area (m^2/g)	Pore volume (cm^3/g)	Average pore radius (nm)
1	Fe_3O_4	480	0.803	1.254
2	$Fe_3O_4@SiO_2$	459	0.784	1.785
3	$Fe_3O_4@SiO_2$-NH_2	390	0.752	1.800
4	**10**	370	0.722	1.825
5	**11**	372	0.722	1.824

General procedure for catalytic oxidation of alcohol to aldehyde catalyzed by 10 and 11

In a 10 mL round bottom flask, 1.0 mmol of alcohol was added to 3.36 g (35.7 mmol) of $DMSO_2$. Then, catalyst (**10** or **11**, 0.013 mol%Pd), was added to the mixture. The flask was equipped with a O_2 balloon (~ 1.0 atm.) and the reaction temperature was adjusted to 120 °C. The reaction progress was monitored by TLC and then GC. The conversion of aldehyde was reported by GC instrument based on pure authentic aldehydes as controls.

Oxidation of alcohol to aldehyde catalyzed by 10 and 11

The catalytic activities of **10** and **11** for the oxidation of benzyl alcohols in the presence of molecular O_2 were studied. Both catalysts showed good to excellent efficiency for all derivatives. The catalysts also showed high selectivity to aldehydes, so that no significant by-product (especially acid) was obtained for any of the derivatives. Significantly, catalyst **11** showed better efficiency and selectivity than **10**. Similarly, catalyst **10** showed much lower efficiencies for coupling reactions (Table S2, entry 1). This effect can be directly attributed to the effect of the presence of the second metal (Ni) in the catalyst, which not only controls the reactivity (increases the selectivity to aldehyde), but also reduces the reaction times and increases the efficiency of the oxidation products with a possible synergistic effect.

Table S2 Oxidation of alcohols to aldehydes catalyzed by $Fe_3O_4@SiO_2$/ (TEMPO)-copolymer-(Chlorophyll *b*) Ni/Pd[a]

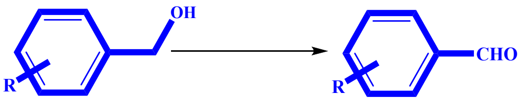

Entry	R	Produc	Time (min)	Conversion (%)[b]/ Selectivity (%)

		t	10	11	10	11
1	H		45	35	95/94	98/99
2	4-MeO		46	40	97/96	99/99
3	4-Me		52	39	94/96	98/99
4	2-Me		44	35	92/96	96/99
5	4-CN		40	20	90/90	95/96
6	4-NO_2		40	35	90/96	96/99
7	1-Naphthyl		55	42	98/99	98/99
8	4-Cl		50	38	85/90	95/96
9	2-MeO		45	40	98/98	99/99
10	4-NO_2,2-Me		52	44	90/96	96/97
11	Nicotin		38	30	94/95	97/97
12	Picoline		44	30	94/95	96/99
13	2-Furfuryl		55	40	80/60	88/77

[a] Reaction conditions: Alcohol (1.0 mmol), catalyst (**10** or **11**, 0.013 mol%Pd), $DMSO_2$ (3.36 g, 35.7 mmol), 120 °C, O_2 balloon (~ 1.0 atm.)

[b] GC yield.

General procedure for Ni/Pd or Pd catalyzed reduction of nitro to amine

In a 10 mL round bottom flask, nitroarene (1.0 mmol), aryl halide (1.0 mmol), catalyst **11** (2.0 mg, 0.026 mol%Pd, 0.068 mol%Ni), $DMSO_2$ (3.36 g, 35.7 mmol), and $NaBH_4$ (2.0 mmol) were mixed and the reaction temperature was adjusted to 120 °C. The reaction was stirred at constant temperature and the progress was monitored by GC instrument.

Reduction of nitro compounds to amines catalyzed by 10 and 11

Then, the activity of catalysts **10** and **11** were studied in order to reduce nitro compounds. Table S3 shows the reduction of nitro compounds (used in the coupling reactions). As shown in the table, reduction of the compounds was achieved in short intervals of 30 to 60 minutes for both catalysts. No significant difference was observed in the performance of the two catalysts and only it seems that the reduction of nitro compounds by catalyst **11** is done in a shorter time.

Table S3 Ni/ Pd catalyzed C-N cross-coupling reaction of aryl halides with amine and nitro precursors[a]

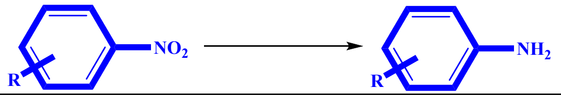

Entry	R	Product	Time (min)		Conversion (%)[b]	
			**10**	**11**	**10**	**11**
1	H		45	40	99	99
2	4-OH		40	36	98	98
3	4-MeO		30	30	98	98
4	4-Me		45	40	96	96
5	4-CN		60	50	95	98

[a] Reaction conditions: Nitroarene (1.0 mmol), aryl halide (1.0 mmol), catalyst (**10** or **11**, 0.026 mol%Pd), $DMSO_2$ (3.36 g, 35.7 mmol), $NaBH_4$ (2.0 mmol), 120 °C.

[b] Based on GC.

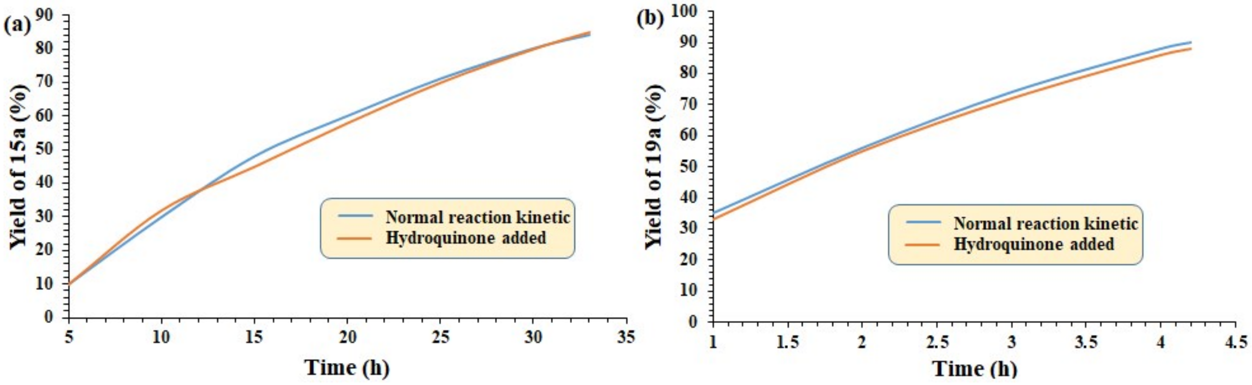

Fig. S10 Influence of hydroquinone as a radical scavenger over the domino (a) oxidation-coupling reaction of benzyl alcohol with phenylboronic, and (b) reduction-coupling reaction of nitrobenzene with iodobenzene

^{1}H-NMR and ^{13}C-NMR results of the coupling products:

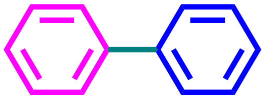

1,1'-Biphenyl (15a, Table 3)

^{1}H NMR (250 MHz, CDCl$_3$) δ: 7.25-7.73 (m, 10H) ppm; ^{13}C-NMR (62.9 MHz, CDCl$_3$) δ: 127.3, 127.5, 130.1, 145.7 ppm [3].

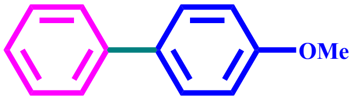

4-Methoxy-1,1'-biphenyl (15b, Table 3)

^{1}H NMR (250 MHz, CDCl$_3$) δ: 3.88 (s, 3H), 6.96 (d, J= 7.5 Hz, 2H), 7.25 (t, J= 7.2 Hz, 2H), 7.44 (t, J= 7.2 Hz, 2H), 7.52-7.58 (m, 3H) ppm; ^{13}CNMR (62.9 MHz, CDCl$_3$) δ: 55.3, 114.6, 127.0, 128.3, 128.2, 128.8, 133.6, 150.0, 159.2 ppm [4].

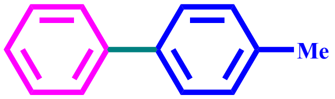

4-Methyl-1,1'-biphenyl (15c, Table 3)

^{1}H NMR (250 MHz, $CDCl_3$) *δ:* 2.52 (s, 3H), 7.36-7.54 (m, 7H), 7.61-7.73 (m, 2H) ppm; ^{13}C NMR (62.9 MHz, $CDCl_3$) *δ*: 21.5, 127.4, 127.9, 128.7, 129.3, 130.1, 131.5, 132.1, 138.0 ppm [5].

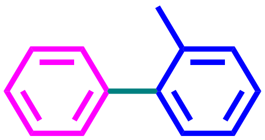

2-Methyl-1,1'-biphenyl (15d, Table 3)

^{1}H NMR (250 MHz, $CDCl_3$) *δ:* 2.44 (s, 3H), 7.40-7.60 (m, 7H), 7.72 m, 2H) ppm; ^{13}CNMR (62.9 MHz, $CDCl_3$) *δ*: 20.5, 125.8, 126.3, 127.7, 128.0, 129.0, 129.1, 130.0, 130.2, 134.8, 141.3, 142.1 ppm [1].

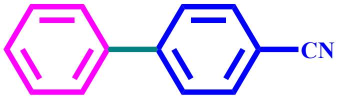

4-Cyano-1,1'-biphenyl (15e, Table 3)

^{1}H NMR (250 MHz, $CDCl_3$) *δ:* 7.44-7.51 (m, 3H), 7.59-7.79 (m, 6H) ppm; ^{13}C NMR (62.9 MHz, $CDCl_3$) *δ*: 110.6, 118.7, 127.4, 128.6, 129.5, 130.0, 132.2, 139.8, 146.4 ppm [4].

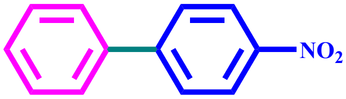

4-Nitro-1,1'-biphenyl (15f, Table 3)

^{1}H NMR (250 MHz, $CDCl_3$) *δ:* 7.42-7.73 (m, 7H), 8.31 (d, *J*= 9.0 Hz, 2H) ppm; ^{13}C NMR (62.9 MHz, $CDCl_3$) *δ:* 125.0, 127.4, 128.0, 128.8, 129.6, 129.9, 141.4, 147.5 ppm [4].

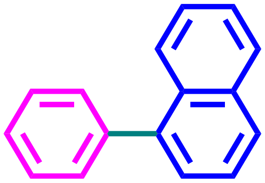

1-Phenylnaphthalene (15g, Table 3)

^{1}H NMR (250 MHz, $CDCl_3$) *δ:* 7.31-7.52 (m, 8H), 7.86-7.95 (m, 3H) ppm; ^{13}CNMR (62.9 MHz, $CDCl_3$) *δ:* 125.4, 125.6, 126.0, 126.2, 126.7, 127.3, 128.3, 128.9, 129.8, 131.4, 133.5, 137.0, 137.6, 140.5 ppm [5].

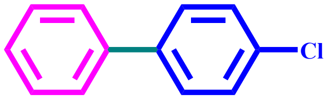

4-Chloro-1,1'-biphenyl (15h, Table 3)

^{1}HNMR (250 MHz, $CDCl_3$) δ: 7.31-7.52 (m, 9H) ppm; ^{13}CNMR (62.9 MHz, $CDCl_3$) δ: 127.0, 127.3, 128.5, 128.5, 128.6, 133.5, 138.2, 139.0 ppm [5].

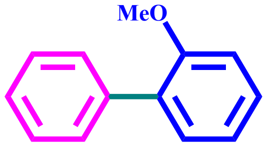

2-Methoxy-1,1'-biphenyl (15i, Table 3)

^{1}H NMR (250 MHz, $CDCl_3$) δ: 3.80 (s, 3H), 6.95-7.02 (m, 2H), 7.31-7.44 (m, 5H), 7.55 (d, *J*= 8.1 Hz, 2H) ppm; ^{13}C NMR (62.9 MHz, $CDCl_3$) δ: 55.7, 111.9, 121.4, 126.5, 128.5, 128.8, 129.4, 131.1, 138.9, 157.0 ppm [6].

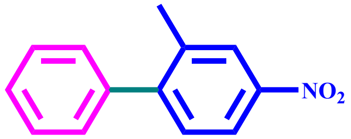

2-Methyl-4-nitro-1,1'-biphenyl (15j, Table 3)

^{1}H NMR (250 MHz, $CDCl_3$) δ: 2.55 (s, 3H), 7.39-7.51 (m, 5H), 7.92-7.88 (m, 3H) ppm; ^{13}CNMR (62.9 MHz, $CDCl_3$) δ: 20.5, 120.9, 125.4, 128.0, 128.2, 128.4, 130.6, 137.1, 139.6, 148.5 ppm [2].

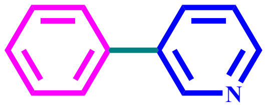

3-Phenylpyridine (15k, Table 3)

^{1}H NMR (250 MHz, $CDCl_3$) δ: 7.35-7.61 (m, 6H), 7.85-7.87 (m, 1H), 8.59 (d, *J*= 4.0 Hz, 1H), 8.88 (s, 1H) ppm; ^{13}C NMR (62.9 MHz, $CDCl_3$) δ: 123.5, 127.0, 128.2, 129.2, 134.4, 136.8, 137.8, 149.3, 149.6 [6].

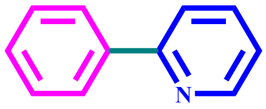

2-Phenylpyridine (15l, Table 3)

^{1}H NMR (250 MHz, $CDCl_3$) *δ:* 7.22 (d, *J*= 9.0 Hz, 1H) 7.43-8.00 (m, 7H), 8.70 (s, 1H) ppm; ^{13}C NMR (62.9 MHz, $CDCl_3$) *δ:* 120.5, 122.2, 126.3, 127.8, 136.8, 139.4, 149.5, 157.2 ppm [4].

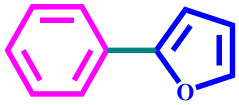

2-Phenylthiophene (15m, Table 3)

^{1}H NMR (250 MHz, $CDCl_3$) *δ:* 6.85 (s,4H), 7.14-6.84 (m, 2H), 7.29-7.23 (m, 5H) ppm; ^{13}CNMR (62.9 MHz, $CDCl_3$) *δ:* 107.2, 119.9, 123.8, 127.4, 127.9, 155.8, 127.1, 143.6 ppm [6].

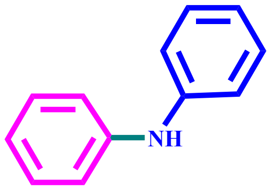

Diphenylamine (19a, Table 4)

^{1}H NMR (250 MHz, $CDCl_3$) *δ:* 6.88 (t, *J* = 7.3 Hz, 2H), 7.14 (d, *J* = 7.7 Hz, 4H), 7.25 (t, *J* = 7.0 Hz, 4H) ppm; ^{13}CNMR (62.9 MHz, $CDCl_3$) *δ:* 117.4, 120.9, 129.2, 143.0 ppm [7].

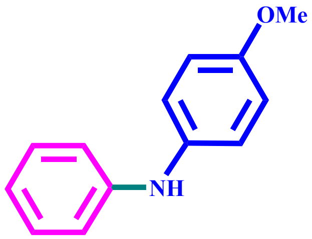

4-Methoxy-*N*-phenylaniline (19b, Table 4)

^{1}H NMR (250 MHz, $CDCl_3$) *δ:* 3.79 (s, 3H), 5.46 (br s, 1H, NH), 6.88 – 6.79 (m, 3H), 6.89 (d, *J* = 7.9 Hz, 2H), 7.14 (d, *J* = 8.1 Hz, 2H), 7.22 (dd, *J* = 8.4, 7.4 Hz, 2H) ppm; ^{13}CNMR (62.9 MHz, $CDCl_3$) *δ:* 55.6, 114.5, 115.6, 119.2, 122.5, 129.0, 135.7, 144.9, 155.6 ppm [8].

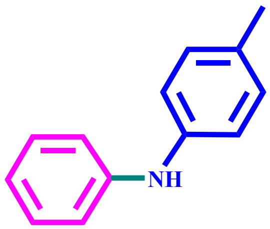

4-Methyl-*N*-phenylaniline (19c, Table 4)

^{1}H NMR (250 MHz, $CDCl_3$) *δ:* 2.31 (s, 3H), 6.86 (t, *J* = 7.3 Hz, 1H), 5.58 (br s, 1H, NH), 7.03-6.97 (m, 4H), 7.13 (d, *J* = 8.1 Hz, 2H), 7.18 (t, *J* = 7.6 Hz, 2H) ppm; ^{13}CNMR (62.9 MHz, $CDCl_3$) *δ:* 20.5, 116.7, 118.4, 120.0, 129.3, 130.4, 140.2, 143.6 ppm [9].

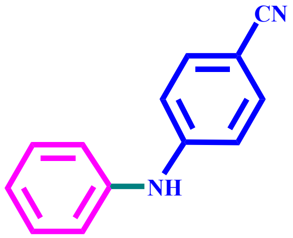

4-Cyano-*N*-phenylaniline (19d, Table 4)

^{1}H NMR (250 MHz, $CDCl_3$) *δ:* 6.96 (t, *J* = 7.3 Hz, 1H), 6.98 (d, *J* = 8.7 Hz, 2H), 7.05 (d, *J* = 8.5 Hz, 2H), 7.22 (d, *J* = 8.4 Hz, 2H), 7.25 (t, *J* = 7.6 Hz, 2H) ppm; ^{13}CNMR (62.9 MHz, $CDCl_3$) *δ:* 118.0, 118.6, 121.4, 125.5, 129.0, 129.4, 141.5, 142.7 ppm [8].

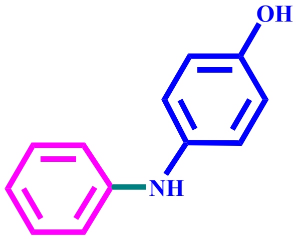

4-Hydroxy-*N*-phenylaniline (19e, Table 4)

^{1}H NMR (250 MHz, $CDCl_3$) *δ:* 6.86 (t, *J* = 7.3 Hz, 1H), 7.03 (d, *J* = 8.5 Hz, 2H), 7.14 (d, *J* = 8.5 Hz, 2H), 7.25 (d, *J* = 8.5 Hz, 2H), 7.20 (t, *J* = 7.3 Hz, 2H), 9.32 (s, 1H) ppm; ^{13}CNMR (62.9 MHz, $CDCl_3$) *δ:* 116.5, 120.2, 122.0, 124..2, 135.4, 142.6, 148.9 ppm [8].

References

[1] Zhuang, X., Zhang, H., Chikushi, N., Zhao, C., Oyaizu, K., Chen, X., & Nishide, H. (2010). Biodegradable and Electroactive TEMPO-Substituted Acrylamide/Lactide Copolymers. *Macromolecular bioscience*, *10*(10), 1203-1209.

[2] Kazemnejadi, M., Shakeri, A., Mohammadi, M., & Tabefam, M. (2017). Direct preparation of oximes and Schiff bases by oxidation of primary benzylic or allylic alcohols in the presence of primary amines using Mn (III) complex of polysalicylaldehyde as an efficient and selective heterogeneous catalyst by molecular oxygen. *Journal of the Iranian Chemical Society*, *14*(9), 1917-1933.

[3] Zhang, J. Q., Cao, J., Li, W., Li, S. M., Li, Y. K., Wang, J. T., & Tang, L. (2017). Palladium/copper-catalyzed arylation of alkenes with N′-acyl arylhydrazines. *New Journal of Chemistry*, *41*(2), 437-441.

[4] Cheng, S., Wei, W., Zhang, X., Yu, H., Huang, M., & Kazemnejadi, M. (2020). A new approach to large scale production of dimethyl sulfone: a promising and strong recyclable solvent for ligand-free Cu-catalyzed C–C cross-coupling reactions. *Green Chemistry*, *22*(6), 2069-2076.

[5] Elazab, H. A., Sadek, M. A., & El-Idreesy, T. T. (2018). Microwave-assisted synthesis of palladium nanoparticles supported on copper oxide in aqueous medium as an efficient catalyst for Suzuki cross-coupling reaction. *Adsorption Science & Technology*, *36*(5-6), 1352-1365.

[6] Dubey, A. V., & Kumar, A. V. (2016). A biomimetic magnetically recoverable palladium nanocatalyst for the Suzuki cross-coupling reaction. *RSC advances*, *6*(52), 46864-46870.

[7] Zhu, X., Zhang, Q., & Su, W. (2014). Solvent-free N-arylation of amines with arylboronic acids under ball milling conditions. *RSC Advances*, *4*(43), 22775-22778.

[8] Kumar, A., & Bishnoi, A. K. (2015). Application of nanoparticle mediated N-arylation of amines for the synthesis of pharmaceutical entities using vit-E analogues as amphiphiles in water. *RSC advances*, *5*(26), 20516-20520.

[9] Nandurkar, N. S., Bhanushali, M. J., Bhor, M. D., & Bhanage, B. M. (2007). N-Arylation of aliphatic, aromatic and heteroaromatic amines catalyzed by copper bis (2, 2, 6, 6-tetramethyl-3, 5-heptanedionate). *Tetrahedron Letters*, *48*(37), 6573-6576.
